# Escalating carbon emissions from North American boreal forest wildfires and the climate mitigation potential of fire management

**DOI:** 10.1126/sciadv.abl7161

**Published:** 2022-04-27

**Authors:** Carly A. Phillips, Brendan M. Rogers, Molly Elder, Sol Cooperdock, Michael Moubarak, James T. Randerson, Peter C. Frumhoff

**Affiliations:** 1 Union of Concerned Scientists, Cambridge, MA, USA.; 2 Woodwell Climate Research Center, Falmouth, MA, USA.; 3 Fletcher School, Tufts University Medford, MA, USA.; 4 Hamilton College, Clinton, NY, USA.; 5 Department of Earth System Science, University of California, Irvine, CA, USA.

## Abstract

Wildfires in boreal forests release large quantities of greenhouse gases to the atmosphere, exacerbating climate change. Here, we characterize the magnitude of recent and projected gross and net boreal North American wildfire carbon dioxide emissions, evaluate fire management as an emissions reduction strategy, and quantify the associated costs. Our results show that wildfires in boreal North America could, by mid-century, contribute to a cumulative net source of nearly 12 gigatonnes of carbon dioxide, about 3% of remaining global carbon dioxide emissions associated with keeping temperatures within the Paris Agreement’s 1.5°C limit. With observations from Alaska, we show that current fire management practices limit the burned area. Further, the costs of avoiding carbon dioxide emissions by means of increasing investment in fire management are comparable to or lower than those of other mitigation strategies. Together, our findings highlight the climate risk that boreal wildfires pose and point to fire management as a cost-effective way to limit emissions.

## Teaser

Boreal wildfires threaten to increase carbon emissions, but fire management presents an opportunity to intervene.

## INTRODUCTION

To limit global average surface temperature rise to 1.5°C above preindustrial levels, emissions of carbon dioxide (CO_2_) must reach net zero by mid-century through a variety of pathways including changes in land use (*1*). Wildfires contribute significantly to losses in forest cover in many biomes (*2*), and hence, changes in fire regimes have the potential to affect global atmospheric CO_2_ concentrations (*2*–*4*). Boreal forests, which cover some 16.6 million km^2^ across the circumpolar region and contain roughly two-thirds of global forest carbon (*5*, *6*), have the potential to play an outsized role in future fire-related emissions. Across the boreal biome, societies manage wildfires to protect human life and infrastructure; however, carbon and climate mitigation are not currently the priorities. Although increasingly widespread boreal wildfires are accelerating the release of carbon stored in these ecosystems (*7*–*9*), neither the impact of these future emissions on policy-relevant carbon budgets nor the potential of fire management to curb them has been quantified.

Wildfires are naturally occurring in North American boreal ecosystems. While these systems have experienced a range of fire severities, including low-severity cultural burns set by Indigenous communities in meadows and grasslands (*10*, *11*), the dominant fire regime of boreal North America is characterized by low-frequency, high severity, crown fires (*12*). The current frequency of fires and area burned, however, exceeds that of historical fire regimes (*4*, *7*). Burned area has nearly doubled in boreal North America in the past 60 years, and the number of large fires (>1000 km^2^) has increased, particularly in western North America (*3*, *8*, *13*). Canada’s boreal forests are more diverse than those in Alaska, both in terms of species composition, fire return intervals, and fire management (*12*, *14*, *15*); however, the risk of fire-mediated increases in greenhouse gas (GHG) emissions remains high across different boreal forest ecoregions. Because of polar amplification, temperatures across the boreal biome are rising at nearly twice the global average rate (*16*, *17*). With further warming, along with more frequent lightning strikes and ignitions (*8*, *18*, *19*), burned area is projected to increase across the circumboreal region over the next several decades (*20*–*22*).

In boreal forests, wildfires generate large quantities of carbon emissions that exacerbate climate warming by combusting organic soil and biomass (*7*–*9*, *23*, *24*). Although fires release carbon as a variety of compounds, including black carbon and methane, the majority of carbon is emitted as CO_2_ (*25*, *26*). Fires also reduce the depth of organic soil that overlays and insulates permafrost, leading to permafrost degradation and thaw, putting large and ancient carbon stores at risk of release to the atmosphere (*27*). Increasing carbon emissions through the projected intensification of fire regimes (*8*, *28*) in boreal forests pose a distinct and unquantified threat to keeping global carbon budgets within levels consistent with meeting the temperature goals of the Paris Climate Agreement.

Management of boreal fires, however, provides an opportunity for intervention and emissions reduction. For 60 years, fires across boreal North America have been managed, primarily through suppression (*29*). The overarching goal of boreal fire suppression is to protect human resources such as life and infrastructure. Notably, limiting carbon emissions is not a current objective of boreal fire management. Here, we assess future carbon emissions from wildfires in North America’s boreal forests and the potential for, and cost-effectiveness of, protecting boreal carbon stores through fire management. Specifically, we (i) estimate the cumulative amount of CO_2_ released from fires across boreal North America between 2020 and 2050, (ii) investigate the efficacy of current fire management practices for limiting fire size, and (iii) assess the cost-effectiveness of boreal fire suppression as a carbon emission reduction strategy. Our analysis of carbon emissions by mid-century addresses the entirety of boreal North America, while our assessment of the cost-effectiveness of boreal fire management as an emissions reduction strategy focuses on Alaska. Our results demonstrate that increased resources for fire management could be a cost-effective strategy for limiting the release of globally meaningful amounts of carbon stored in boreal forests.

## RESULTS

### Boreal North America CO_2_ emissions by mid-century

**Fig. 1.**
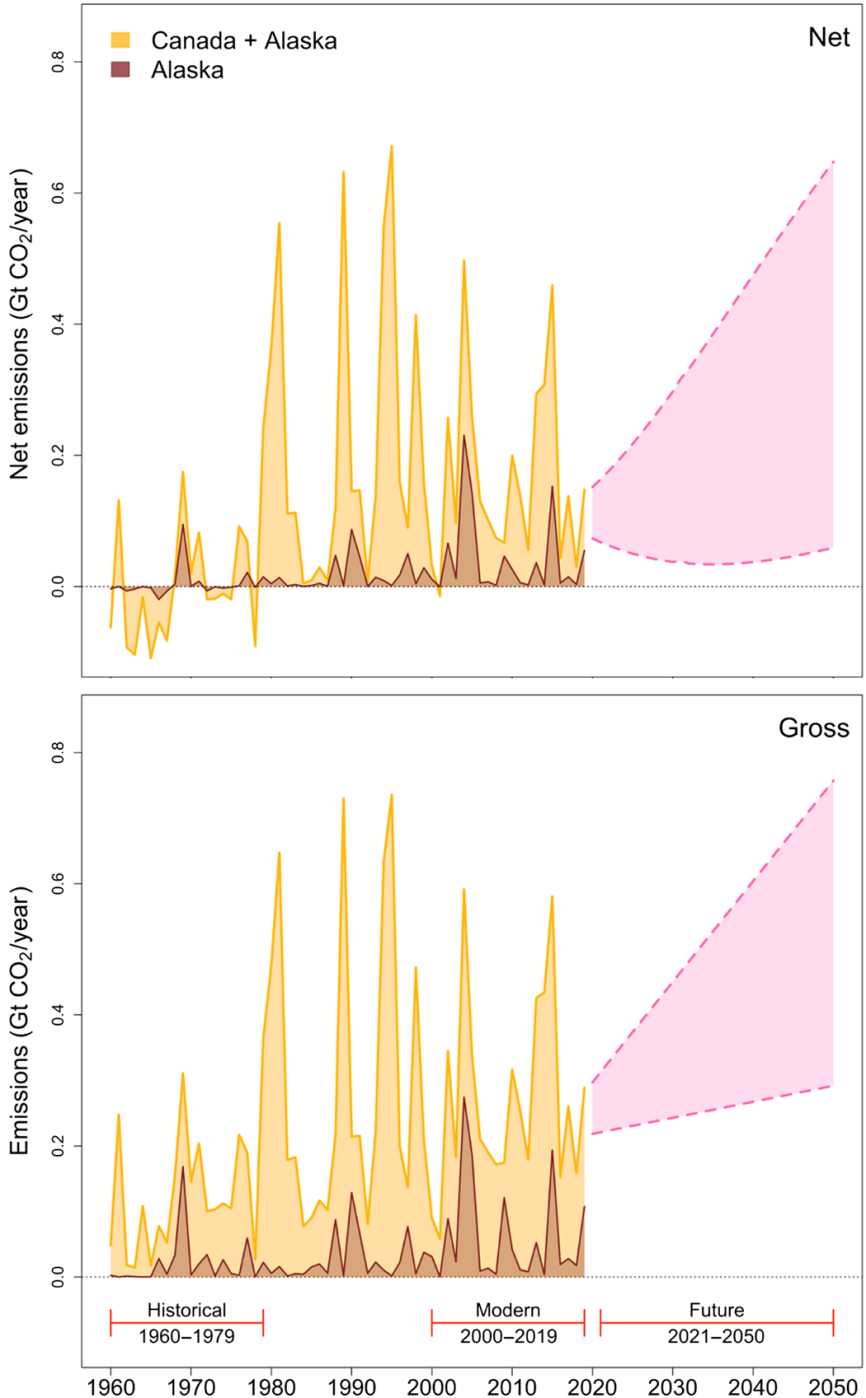
Observed (1960–2019) and projected (2020–2050) gross and net CO_2_ emissions from boreal North America. Brown areas represent emissions from boreal Alaska, while orange areas represent combined emissions from boreal North America (both Alaska and Canada). Pink areas show the range of projected average annual emissions for boreal Alaska and Canada through mid-century.

Our synthesis of the published literature indicates that burned area is projected to increase by 24 to 169% from 2020 to 2050 in Alaskan and 36 to 150% in Canadian boreal forests (Fig. 1). When combined with our estimates of net CO_2_ emissions, these results suggest that wildfires in boreal North America are projected to cumulatively release net emissions between 1.33 and 11.93 Gt of CO_2_ if current levels of fire suppression are maintained through mid-century. Annual emissions average to roughly 0.2 Gt of CO_2_ across boreal North America, although emissions consistently increase over time (detailed breakdown available in table S1). Similarly, enhanced fire management could avoid the release of a conservatively estimated 0.89 to 3.87 Gt of CO_2_ between 2021 and 2050.

### Fire management impacts on burned area in Alaska

**Fig. 2.**
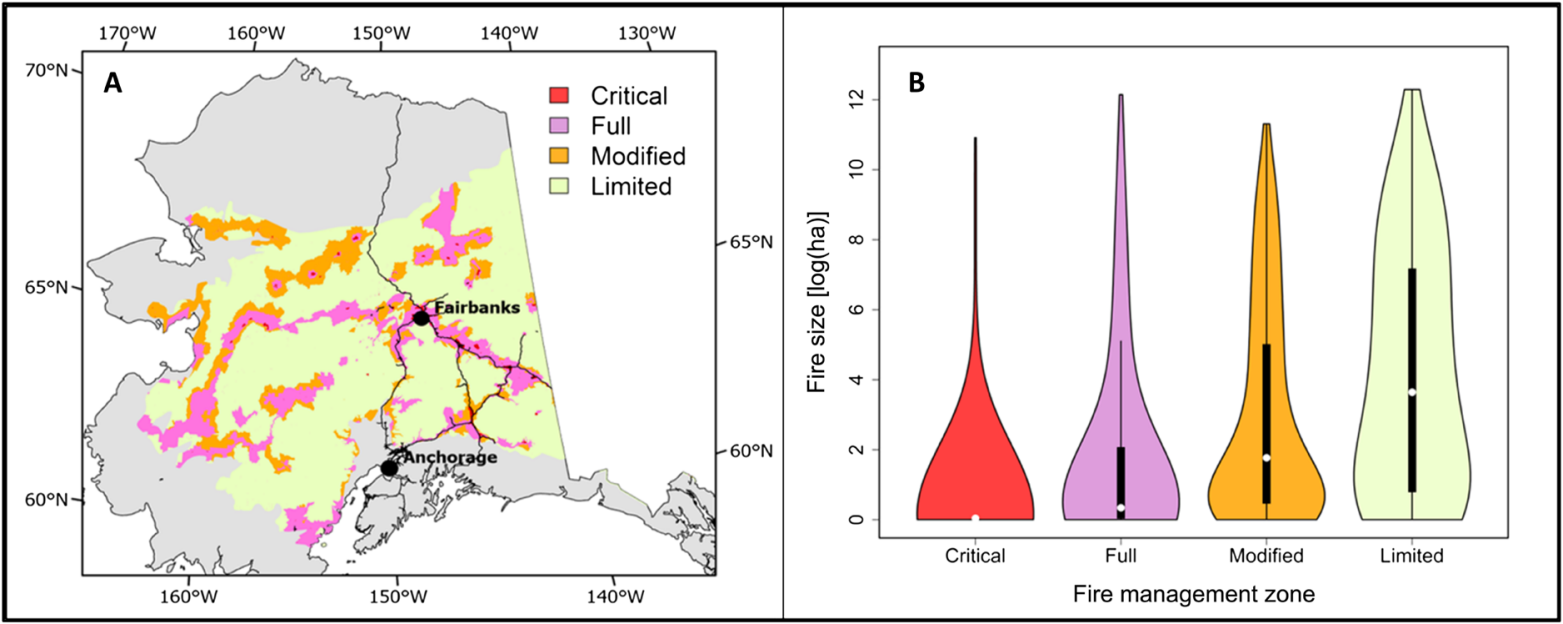
FMZs in boreal Alaska and the violin plots showing the median, range, and distribution of fire size for each FMZ between 2000 and 2018. (**A**) Map of Alaska detailing current FMZs throughout our study region. Critical areas receive the greatest fire suppression effort, while limited areas receive the least. Fires in full and modified zones receive progressively less fire suppression effort compared to critical zones (details in the Supplementary Materials). Fires in limited areas are often monitored and left to burn. Gray areas represent those outside our study area. (**B**) Violin plots for fires between 2000 and 2018 on a logarithmic scale. White points indicate median fire size across each management zone. Black rectangles represent the interquartile ranges, and black lines represents the data distribution between the upper and lower adjacent values (first and third quartiles ±1.5 times the interquartile range, respectively). The polygons show the full range of values, while the width of each polygon represents the frequency of the values. Note the highly compressed interquartile and value ranges in the critical polygon.

Individual fires are, on average, smaller in zones, receiving greater suppression effort relative to lower suppression zones after taking into account other environmental predictor variables (Fig. 2B and fig. S1B). The results from our linear model suggest that fire management zone (FMZ) is an important predictor of burned area, explaining ~22% of the total variability in final fire size (*F*_3,3407_ = 335.42, *P* < 0.001). Our random forest model yielded an average back-transformed, cross-validated *r*^2^ of 0.43. The final predictor subset for our random forest regression involved 11 variables including vegetation, fuels, weather, cause, fire year, and FMZ. Our assessment of conditional variable importance (*30*) illustrated that, although factors like vegetation composition and fire weather are important, FMZ at the point of origin was the fifth most important predictor of fire size, with only maximum temperature, mean temperature, maximum duff moisture code, and fire cause ranking as more important predictors. Partial dependence plots indicate a negative relationship between suppression effort (represented by management zone) and fire size (fig. S1). These findings are consistent with past work showing that machine learning models predicting the final fire size at the time of ignitions, as well as trained on observations from limited fire suppression zones, overpredict the final fire size in areas targeted for more intensive fire management (*31*).

### Economic results

In our primary model specification, we found that, on average, a 1% increase in total spending reduced fire size by 0.21 ± 0.10% (tables S2, column 2, and S3), indicating that increases in expenditures have the potential to reduce the burned area. Alternate specifications (table S4)—which test the model’s robustness to different fire costs, different approaches to zero-cost fires, and the exclusion of human caused ignitions—yielded similar results. We also found no significant difference in the effect of spending on fire size in areas with and without roads (see Supplementary Methods). The presence of roads is highly correlated with FMZ designation, thus reducing the strength of FMZ as an instrument (with roads: *F*_3,132_ = 6.65, *P* = 0.86; without roads: *F*_3,624_ = 17.88, *P* < 0.08).

We found the average cost of avoiding 1 metric ton of CO_2_ emissions to be $12.63 [standard error range of $8.78 to $22.52 and a 95% confidence range of $6.79 to $90.76 (fig. S2); all economic results are expressed in 2015 USD, while future projections are expressed in 2020 USD]. Emissions from fire management (i.e., burning of fuel for airborne suppression/engines), for the 8 years for which we have cost data, represented an average of 0.57% of annual net emissions from wildfires (table S5).

Using aggregated cost data, we found the average fire management cost of an Alaskan fire season is approximately $133 million in 2020 USD, a small percentage of which goes toward the direct response costs. An investment of, on average, $696 million/year (in 2020 USD) over 2021–2030 (or roughly $6.96 billion in total) would be needed to keep carbon emissions from Alaska wildfires at historical levels over the next decade (see Supplementary Methods). Over the next 30 years, reducing emissions to historical levels would require an investment of between $7.1 billion and $50 billion.

## DISCUSSION

Our results show that wildfires in boreal North America present both substantial risks of increasing carbon emissions and, specifically in boreal Alaska, substantial opportunities to cost-effectively limit those emissions through increased fire management. Thus far, limiting boreal wildfires has not been explicitly considered as a climate mitigation strategy. While calculations of avoided emissions inherently include uncertainty in predicting future events, our results suggest that increased investments in boreal fire management should be considered within the portfolio of climate mitigation strategies needed to bring emissions to net zero by mid-century and limit global average temperature rise to 1.5°C, the aspirational target set by the Paris Climate Agreement.

Across boreal North America, burned area has nearly doubled over the past few decades, a marked departure from fire regimes that historically governed the carbon balance of these ecosystems (*4*). Left unchecked, our results suggest that wildfires in boreal Alaska and Canada may release a net of 1.33 to 11.93 Gt of CO_2_ between 2020 and 2050 or, conservatively, 0.33 to 2.98% of the remaining global carbon budget for limiting global warming to 1.5°C (table S6).

Although these conservative estimates of net emissions (1.33 to 11.93 Gt of CO_2_) incorporate CO_2_ uptake from postfire regrowth by mid-century, they do not account for several ecosystem processes that would increase net emissions and the warming impact from boreal fires. For instance, wildfires can accelerate permafrost thaw (*21*, *27*, *32*), exposing anciently stored carbon to decomposition by soil microbes. While this can happen gradually, fire may also contribute to nonlinear, abrupt thaw with even greater carbon consequences (*33*). By some estimates, emissions from postfire decomposition over a 100-year period are more than six times the amount of soil carbon lost to combustion (*34*). Our analysis also did not include emissions of other GHGs emitted from fires such as CH_4_ and N_2_O, which amplify radiative forcing (*35*, *36*). In contrast, a shift toward deciduous-dominated forests may reduce the likelihood of future burns, as they are less flammable than the spruce forests they replace (*37*–*39*). These transitions may both increase carbon storage while also exacerbating permafrost thaw (*40*). Similarly, fire-mediated changes to land surface albedo can facilitate local cooling in boreal regions (*35*), as snow cover in recently burned areas increases reflectivity. An increase in suppression activities may reduce these effects; however, they are largely regional and seasonal (*41*), while GHG impacts from boreal fire emissions are global and year-round. As a result, our conservative estimate of boreal fire emissions likely underestimates the true carbon consequences of an intensifying boreal fire regime.

Our analyses reveal that current fire suppression activities in Alaska, on average, reduce fire size and thus emissions in areas that receive the greatest protection (Fig. 2). This is despite the fact that current management efforts largely do not aim to minimize fire size. Rather, current fire management practices primarily aim to contain and control fires to protect human life, residences, and infrastructure. In some cases, these strategies manifest in an effort to contain the fire to as small an area as is possible, especially during initial attack (*29*). Although predicting the influence of management on any single fire is complex and necessitates more detailed, local-scale modeling, our analysis suggests that, if reducing fire size (and carbon emissions) was an explicit goal and greater resources were allocated toward this end, suppression would likely be even more effective in limiting emissions than indicated by our analyses.

An increased focus on initial attack may be particularly effective in limiting emissions, as peaks in fire ignitions tend to precede peaks in burned area by several weeks to over a month (*8*), and the majority of initial attack failures is due to response time (*42*). Overwintering fires may also represent an opportunity for initial attack, as these fires are associated with high carbon emissions and generally flare up very early in the fire season when resources are otherwise available (*43*). Further, focusing resources on carbon-rich and high wildfire risk areas such as peatlands could also limit emissions.

Our study also underscores the importance of weather in driving fire behavior and size (*31*), highlighting one of the many consequences of continued warming at high latitudes. Maximum and mean temperatures over the duration of a fire were the two most important variables in our model of individual fire size, reinforcing the urgency of reducing global emissions from all sources to limit temperature increases and the resulting intensification of boreal fire regimes. Warmer surface temperatures can drive increases in burned area by drying out vegetation and soils in boreal forests, priming them to burn (*3*, *44*), and increases in atmospheric energy are associated with more lightning strikes (*8*, *19*). These trends illustrate how, without intervention, predicted global temperature increases, which are amplified at high latitudes, will continue to increase the burned area.

Further, our economic analyses indicate that increasing fire suppression expenditures in boreal Alaska would decrease fire size and emissions. Between 2007 and 2015, increasing expenditures by 1%, on average, reduced fire size in boreal Alaska by 0.21%, regardless of an ignition’s proximity to a road. At an average of $12.63/metric ton of CO_2_, the direct cost of reducing net emissions through boreal wildfire management in Alaska compares favorably to other CO_2_ mitigation measures (Table 1). While this cost estimate does not account for emissions from fuel used during fire management, these emissions are relatively modest, constituting less than 1% of average annual CO_2_ emissions from Alaskan boreal wildfires (table S5). Therefore, despite the emissions from fire management itself, targeted suppression in boreal Alaska appears to be a cost-effective way to reduce emissions.

**Table 1.** Estimates of static, direct costs of CO_2_ emissions reduction and negative emissions technologies and approaches. Ranges for fire management represent a 95% confidence interval. Values for other approaches and technologies represent the range of estimates depending on land availability, scale of implementation, and market demand.

**Technology or approach**	**Cost (USD/metric ton of CO_2_)**
Power sector technologies*	
Onshore wind	23–26
Utility-scale solar photovoltaic	32–41
“Advanced” nuclear	58
**Negative emissions technologies** **and approaches^†^**	
Coastal blue carbon	0.75–30
Soil carbon sequestration	0–50
Direct air capture	90–600
Afforestation, reforestation, forest management	15–50
**Fire management**	
Boreal fire management in Alaska	6–91

*Estimates compare the cost per metric ton of CO_2_ abated by replacing electricity generated from coal or natural gas with electricity generated by a cleaner alternative (*80*, *81*).

^†^Estimates represent current costs per metric ton of CO_2_ sequestered for implementation at scale (*82*).

To limit ecological impacts, increased fire management could be designed to help reestablish historical fire regimes in Alaska’s boreal forests. Although boreal forests evolved with fire, recent fire regimes far exceed those of previous decades when the influence of anthropogenic climate change was negligible (*45*). Concerns about the ecological impact of fire suppression are widespread, partly due to a century of suppression, fuel build up, and subsequent fires in temperate forests across western North America. However, the historical fire regimes of these geographies, characterized by high-frequency, low severity surface fires, are fundamentally different from the low-frequency, high-severity regimes in North American boreal forests. Suppression efforts in the western United States effectively eliminated natural fire regimes, lengthening fire return intervals, increasing fuel loads and landscape connectivity (*46*), and increasing the likelihood of catastrophic high-intensity crown fires (*47*). In contrast, high-intensity crown fires are characteristic of North America’s boreal fire regime, particularly in areas dominated by black spruce (*48*). Fuel treatments, similar to those used in temperate forests of the western United States, may not modify and, in some cases, may actually increase carbon loss when implemented in boreal forests (*49*, *50*). Conversely, these same treatments are an important climate adaptation tool in boreal communities, where fuel modification can temper fire behavior and enhance defensibility during low to moderate fire weather (*51*, *52*). On a landscape scale, however, these treatments would be incongruent with the historical occurrence of fire in these ecosystems. Thus, increased fire management in boreal forests should be designed to not eliminate fire on the landscape but to support community adaptation and allow fire regimes to return to historical levels while society quickly moves to net zero carbon emissions.

In boreal Alaska, enhancing fire suppression efforts to limit wildfires to historical levels would avoid the release of a conservatively estimated 0.89 to 3.87 Gt of CO_2_ between 2021 and 2050. We estimate the annual response costs for fire suppression at this scale in boreal Alaska at $696 million/year, on average, through 2030; costs that might be applied to both enhance permanent personnel and develop infrastructure to limit fires in more remote areas. This would entail a sizeable increase in fire management budgets for Alaska, where state and federal fire management expenditures jointly average about $133 million/year, a small percentage of which go toward direct response costs. Estimates of these annual average response costs vary from federal costs of $27 million to total costs of $85 million (*53*). However, it is also small relative to overall U.S. federal expenditures in wildfire suppression, which totaled over $3.1 billion in 2018. Within the United States, Alaska receives a disproportionately small amount (less than 4% on average) of federal resources for fire management (Suppression Costs, National Interagency Fire Center, R. Jandt) despite accounting for roughly 20% of the U.S. land area and half of the average annual U.S. fire emissions (fig. S3). While more research is required to understand an optimized allocation of resources and appropriate implementation tactics, our results show that, over the next 10 years, reducing emissions to that of historical regimes in Alaska would require an average investment of roughly $696 million annually (cumulatively about $6.96 billion) and prevent the release of 0.89 to 3.87 Gt of CO_2_.

Beyond carbon mitigation, increased investments to limit boreal wildfires to historical levels would yield multiple additional benefits. Increased fire management would limit particulate matter 2.5 (PM2.5) emissions that reduce air quality and lead to serious health consequences including asthma, pneumonia, and cardiovascular diseases. Health risks are particularly heightened for vulnerable populations including children and the elderly (*54*) and extend both to adjacent communities and, during severe wildfire seasons, communities at considerable distance, including in the conterminous United States (*55*). Increased fire management would also provide additional opportunities for employment (*56*) and could minimize disruptions to certain subsistence activities (*57*). Thus, in addition to climate benefits, enhanced fire management could bring a broad array of ecosystem services, including health, economic, and ecological benefits to boreal communities.

The analyses presented here underscore the magnitude of carbon emissions from North American boreal forests and what can be avoided through the enhanced fire management in Alaska through mid-century. While more research is required to determine the feasibility in the distinct and provincially variable Canadian management context (*14*), similarly, enhanced fire management in boreal Canada could avoid the release of an estimated 2.95 to 8.41 Gt of CO_2_ by 2050. On a global scale, Eurasia, as well as Siberia in particular, contains the majority of Earth’s boreal forests. Fires in this region often kill fewer trees and burn less intensely than those in North America (*12*). Further work is needed to determine whether available data on fire carbon emissions, fire management priorities, and expenditures would enable an expansion of this analysis to the full circumboreal region.

Together, our results indicate that boreal fire management in Alaska and perhaps beyond is a notable, cost-effective, and previously overlooked climate mitigation strategy, one that should be taken up within a broader portfolio of action to bring global carbon emissions across sectors toward net zero by mid-century. We ignore these fires at our peril. Now is the time to accelerate both our understanding and practical implementation of fire management as a priority strategy to keep the vast stores of boreal forest carbon in the ground.

## MATERIALS AND METHODS

We first used datasets of historical fire occurrence, carbon emissions, and projections of future burned area in Alaska and Canada to bound the magnitude of carbon loss from boreal wildfires by 2050. Using available data from Alaska, we developed a statistical model of individual fire size as a function of, among other variables, FMZs of differing levels of suppression effort to determine whether current practices are effective in constraining burned area. Last, we analyzed suppression expenditure data to evaluate the relationship between spending and fire size and to quantify the cost per metric ton of averted CO_2_ emissions.

### Projecting carbon emissions from wildfires in boreal North America

To determine the scope of wildfire emissions across boreal North America, we conducted literature searches for projections in burned area for both Alaska and Canada that yielded 464 and 1394 papers, respectively. We narrowed our initial search to 13 papers for Alaska and eight for Canada that met our requirements of projecting burned area over the entirety of each respective region (table S6; detailed methods in the Supplementary Materials) and further searched the references and citing articles of the selected papers to ensure we did not overlook relevant studies (*8*, *20*–*22*, *28*, *34*, *37*, *53*, *58*–*64*, *74*–*78*). All papers came from peer-reviewed journals.

For each paper identified, we extracted the start and end date of projections, as well as the percent increase in burned area over that time period. If projected increases by 2050 were not specified, then we estimated this value by assuming a linear increase over the course of each projection [similar to trends in (*28*, *34*, *58*)]. We used the upper and lower quartiles of these projections for each region to conservatively bound our estimates of burned area by mid-century (table S7).

#### CO_2_ budgets

To characterize the remaining global CO_2_ budget for a 67, 50, and 33% probability of keeping global surface temperature rise below 1.5° and 2°C, we used emission budget estimates from the most recent Intergovernmental Panel on Climate Change calculations of allowable remaining net emissions (table S6) (*79*).

#### CO_2_ emissions

To estimate combustion (carbon emissions per unit burned area) for boreal wildfires, we averaged field measurements from 548 burned sites in Alaska, Northwest Territories, and Saskatchewan, resulting in an average combustion rate of 3.325 ± 1.818 kg of C/m^2^ (*65*). We used this value to translate historical, modern, and future burned area into carbon emissions measured in gigatonnes (1 billion metric ton).

To translate carbon emissions into CO_2_ emissions, we used a boreal-specific emissions factor derived from Akagi *et al.* (*26*). Because emission factors are presented relative to dry matter combustion, we calculated the relative proportion of CO_2_ emissions compared to other carbon-containing compounds, adjusted by molecular weight, resulting in a carbon-specific emission factor of 0.84 for CO_2_. We also used the emissions from historical fire regimes to contextualize current and projected increases. The period from 1960 to 1979 was defined as a historical baseline, 2000 to 2019 as modern fire regimes, and 2021 to 2050 as our future period (fig. S4).

To determine the extent to which ecosystem regrowth counteracts wildfire emissions, we estimated annual net ecosystem productivity in boreal forests after fire [(*35*, *66*) and fig. S5], such that carbon sequestration did not begin until approximately 14 years after fire, and over a 150-year period, carbon accumulation roughly equaled the initial pulse of wildfire emissions (3.325 ± 1.818 kg of C/m^2^). By accounting for carbon dynamics as a function of time since burn, we calculated net emissions at 2050 for fires that burned between 1960 and 2019 and projected emissions to 2050.

### Assessing the relationship between fire management and burned area in Alaska

#### Study area

We focused this analysis on Alaska due to data availability and consistency across the state in fire management operations. Fires throughout boreal Alaska represent the majority of both burned area (roughly 94% of burned area from 2000 to 2018) and current suppression efforts for the state. To capture these, we defined our study region using U.S. Environmental Protection Agency–defined level I ecoregions to include Taiga and Northwestern Forested Mountains [(*62*) and fig. S6]. To capture fires that started outside but spread into our study area, we created a 3.2-km buffer (2 miles) around these areas (Fig. 2A). All other data were clipped to this region.

#### Fire history data

We assembled burned area data from the Alaska Interagency Coordination Center (AICC) and the Canadian National Fire Database (*68*). These data include information about each individual fire’s point of origin, year and month of burn, size in hectares, management zone (for fires after 1985), and general cause. We limited the Alaska-specific portion of our study to fires that occurred between 2000 and 2018 and further filtered our data to focus on fires that were discovered between 1 June and 31 August of any given year when ignitions that drive the majority of area burned occur (*8*, *69*). Our final dataset contained 3411 fires (further details are available in the Supplementary Materials).

#### Fire cause

To account for differences in fire size based on ignition source (*69*), we included the cause of the fire as a predictor in our model. Human-caused fires are typically smaller than those ignited by lightning because they are often closer to roads, easier to detect, and often occur in areas that receive the most suppression resources. We classified ignition source of fires into three categories: human, lightning, and undetermined ignition source (table S8).

#### Vegetation data

To account for the role of vegetation in burned area, we extracted data from the U.S. Department of Agriculture’s LANDFIRE product at 30-m resolution (*70*). We first masked the “unburnable” vegetation categories as outlined by the Alaska Fire Service (table S9) and grouped the remaining vegetation types into five groups: black spruce (*Picea mariana*), white spruce (*Picea glauca*), deciduous, grass, and a general burnable category. For a given fire’s aerial extent, we calculated the proportion of these vegetation classes contained within its boundaries. For fires larger than 100 ha, we used spatial extents from the AICC fire polygon data. For those smaller than 100 ha (for which spatially explicit data are inconsistently recorded for the years of our study), we created a circular buffer around the point of origin that matched the fire’s final size.

#### Fire weather data

To ensure we captured weather conditions preceding and during a fire, we calculated the daily maximum and mean of several fire weather indices (table S10) over the duration of each individual fire from the Canadian Forest Fire Weather Index using Modern era retrospective analysis for research and applications, version 2 (MERRA-2) reanalysis from version 2.5 of the Global Fire Weather Database (*71*, *72*). We calculated the duration of each fire using the difference between the date of discovery and extinguishment.

#### Fire management zones

To facilitate rapid decision making, all land across Alaska is grouped into one of four FMZs (Fig. 2A) that prioritize fire suppression efforts on a continuum (Critical-Full-Modified-Limited). These designations integrate several values at risk in any given area, including human life, structures, and wildlife and streamline the decision-making process across government agencies, native communities, and individuals that own and manage the land. Notably, risk of carbon release is not currently incorporated into current Alaska fire management priority-setting considerations. Fires in critical zones receive the most aggressive suppression, while fires in limited zones are often monitored without intervention. Full and modified zones receive intermediate suppression effort (further details are available in the Supplementary Materials). Although these designations cannot capture the full range of considerations involved in decision-making, they serve as a proxy for suppression effort and thus provide a valuable way to evaluate the effectiveness of fire management. Although nonstandard responses may obfuscate the relationship between FMZ and fire size, less than 15% of total individual fires in 2019, the first year for which these data were reported, were managed this way (2019 Fire Data, AICC), suggesting a tight relationship between FMZ and response.

#### Model parameterization

We first used a linear model to predict individual fire size as a function of FMZ. To understand the role of FMZ within the context of other drivers, we used a random forest regression to predict fire size as a function of vegetation, weather, cause, fire year, and FMZ (detailed methods available in the Supplementary Materials). Because of the distribution of our data, we log-transformed fire size in hectares burned before running our models. Our model contained 500 trees with three variables selected at each node split and was cross-validated with 80% of the data used for training and 20% for testing. We interpreted our models using variable importance and used summary statistics to illustrate patterns. To understand variable importance, we used a conditional inference random forest in the party package [R version 3.5.1; (*30*)]. Analyses and data manipulation were performed in R (version 3.5.1), Google Earth Engine (*73*), and ArcGIS Pro (version 2.4.2).

### Quantifying costs associated of limiting burned area and averting CO_2_ emissions

#### Economic data

To estimate both the cost of fire management on a per area basis and the cost of averting a metric ton of CO_2_ emissions, we compiled the best available data from the Bureau of Land Management (BLM) detailing expenditures on 857 individual fires between 2007 and 2015 (see the “Datasets” section in the Supplementary Materials), including both BLM costs and the percentage of land that received BLM protection. This dataset included fires in areas under both the State of Alaska and Federal fire protection (fig. S7). We combined these data to estimate the total costs of each fire. Total costs are approximately equal to BLM costs divided by the fraction of the burned hectares under BLM protection, except in limited zones, where all costs are covered by the BLM. We excluded data for fires in which reported BLM area exceeded the total area by greater than 1% and fires that fell outside the geographic range of our study (32 fires). Our final dataset contained 825 fires.

To estimate the future costs of suppression, we used aggregated data of total suppression expenditures (detailed methods in the Supplementary Materials). We chose those years based on data availability, and annual fire season expenditures ranged from roughly $40 million to $303 million (BLM Alaska Fire Service; dataset in the Supplementary Materials).

#### Modeling suppression costs

Our goal was to estimate the effect of increased management spending on individual fire size. To control for potential endogeneity in the relationship between fire size and suppression costs, we used a two-stage instrumental variable (IV) approach with FMZ as our instrument (detailed methods in the Supplementary Materials). IVs must satisfy the relevance and excludability restrictions; in this case, FMZ must be a good predictor of cost and only affect the burned area through the channel of cost. Table S2 gives the first stage results, which show that FMZ is a significant predictor of cost, satisfying the relevance restriction. Traditionally, the excludability restriction is more challenging to fulfill. For this analysis, FMZ is assigned before a fire starting, so it cannot be affected by the fire size the way cost is, avoiding the reverse causality problem that motivates the use of an IV. In the first stage, we estimated the total cost using FMZ and the control variables, which were selected using least absolute shrinkage and selection operator (LASSO) (detailed methods in the Supplementary Materials). FMZ is a significant predictor of cost (*F*_3,790_ = 33.41, *P* < 0.0001), thus exceeding the weak instrument threshold (table S2). In the second stage, we estimated the burned area using the modeled costs, which were then free from reverse causality issues (table S2, column 2). The alternate specifications (table S4, columns 1 to 4; BLM costs instead of total costs, only fires caused by lightning, adding 0.01 instead of 1 to costs before taking logs, and using an inverse hyperbolic transformation) yielded similar results.

#### Calculating the price of averted emissions

Using results from the above analysis, average per-hectare CO_2_ emissions and the costs and total area of each fire, we constructed a hectare-weighted average cost per metric ton of CO_2_ emissions avoided using the equation below, where *A_i_* is the hectares burned in fire *i*, *C_i_* is the management cost of that fire, *e_i_* is the rate of emissions per hectare burned in fire *i*, and β is the regression coefficient expressing the average percent decrease in the burned area from a 1% increase in expenditures

$$\text{Cost per metric ton of}\;{\text{CO}}_{2}\;\text{emissions avoided}=\frac{A_{i}}{\sum A_{i}}\sum_{i=1}^{n}\frac{C_{i}}{A_{i}}\frac{1}{\beta e_{i}}$$

To capture the distinct challenges of fighting wildfire in remote areas and the way in which those challenges may modify suppression costs, we also evaluated how the cost of averting emissions change in fires that ignite near roads and those in roadless areas (detailed methods in the Supplementary Materials).

#### Estimating CO_2_ emissions from fire management

The use of jet fuel and gasoline during fire suppression also produces CO_2_ emissions. To account for this, we estimated emissions from fuel use using fire suppression costs, average fuel prices, and CO_2_ emission coefficients. In the absence of data on fuel expenditures and to avoid underestimating the emissions from fuel use during fire suppression, we explored an upper-bound scenario in which 25% of total expenditures went toward the fuel, and these funds were evenly split between jet fuel and gasoline. We then used the price of gasoline and jet fuel from July of each target year [real petroleum prices, US Energy Information Administration (USEIA)] to estimate the liters of fuel purchased and used an emission coefficient to calculate the total CO_2_ emissions (carbon dioxide emission coefficients, USEIA).

## Supplementary Materials

### This PDF file includes:

Figs. S1 to S7 Tables S1 to S10 Supplementary Methods Datasets References
