# Supplementary material for: Escalating carbon emissions from North American boreal forest wildfires and the climate mitigation potential of fire management
Source: Sci Adv. 2022 Apr 27;8(17):eabl7161. doi: 10.1126/sciadv.abl7161 (PMC9045718; doi:10.1126/sciadv.abl7161)
Supplement: Supplementary file 1 — Figs. S1 to S7 Tables S1 to S10 Supplementary Methods Datasets References [file sciadv.abl7161_sm.pdf]

Science Advances
AAAS

Supplementary Materials for

Escalating carbon emissions from North American boreal forest wildfires and the climate mitigation potential of fire management

Carly A. Phillips*, Brendan M. Rogers, Molly Elder, Sol Cooperdock, Michael Moubarak, James T. Randerson, Peter C. Frumhoff

*Corresponding author. Email: carlyphillips@uvic.ca

Published 27 April 2022, *Sci. Adv.* **8**, eabl7161 (2022)
DOI: 10.1126/sciadv.abl7161

This PDF file includes:

Figs. S1 to S7
Tables S1 to S10
Supplementary Methods
Datasets
References

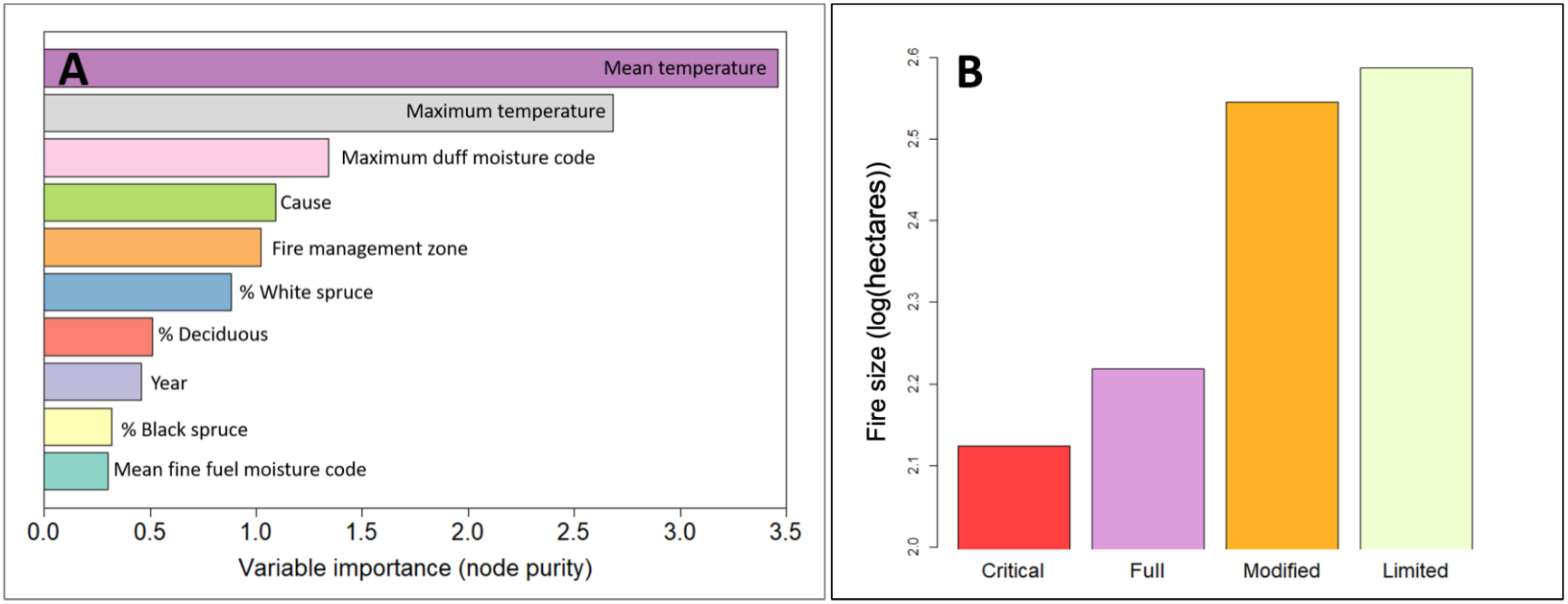

Figure S1: Panel A) Conditional variable importance for predicting fire size from our random forest model using the party package (*30*). Panel B) Partial dependence plots from random forest showing the relative fire size across fire management zones in Alaska's boreal forests.

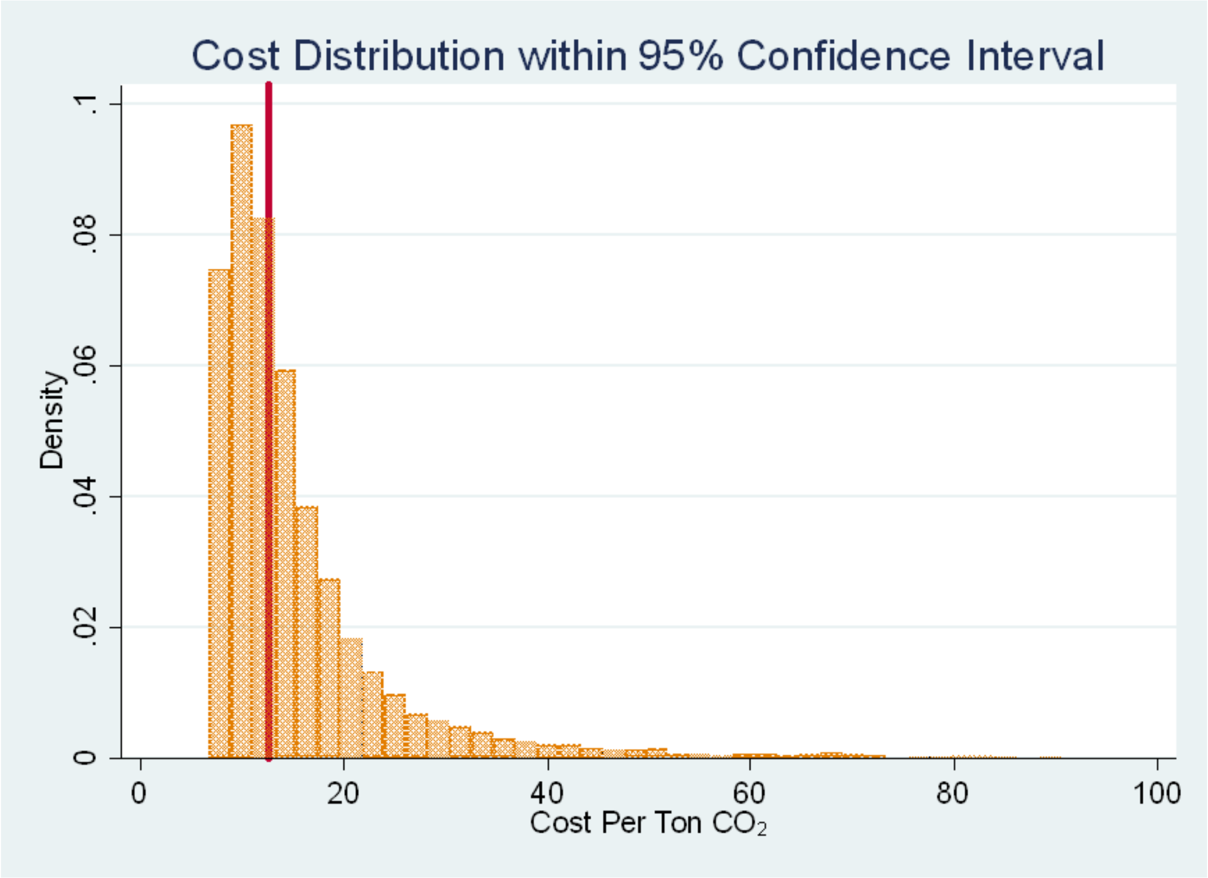

Figure S2: Simulated probability density function of the cost per ton of CO_2 emissions avoided, using 10,000 observations, mean, and 95% confidence interval of the regression coefficient. We simulated a normal distribution using the mean and standard deviation of the regression coefficient, and then each point was transformed according to the cost per ton equation in the text. The average cost of $12.63 is highlighted by the vertical bar.

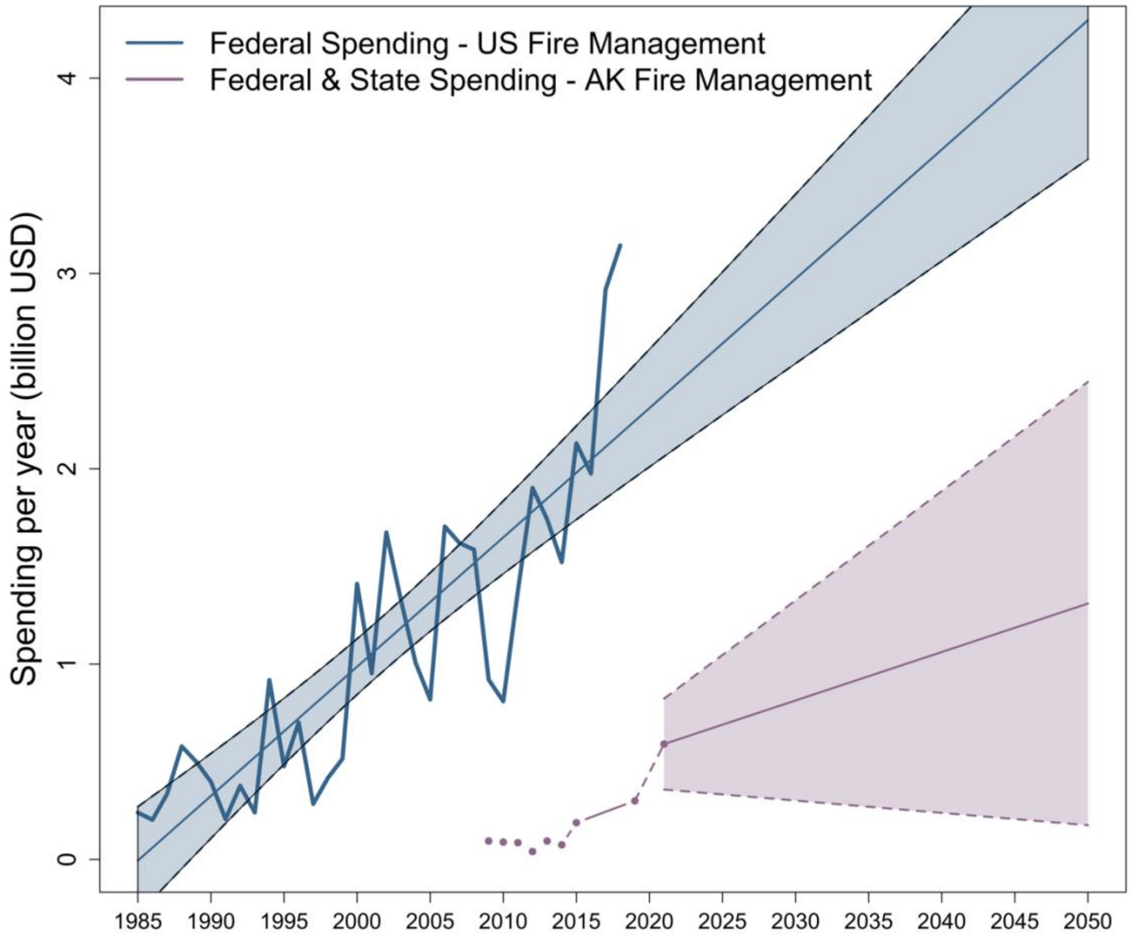

Figure S3: Blue line indicates annual federal expenditures for wildfire management across the United States from the National Interagency Fire Center. Blue shaded area represents the modeled relationship of spending and time with 95% confidence intervals, and projected to 2050. Purple line indicates annual federal and state expenditures for wildfire management in Alaska. Purple shaded area represents estimated range of expenditures in AK wildfire management needed to reduce projected CO_2 emissions to historical levels (1960-1979).

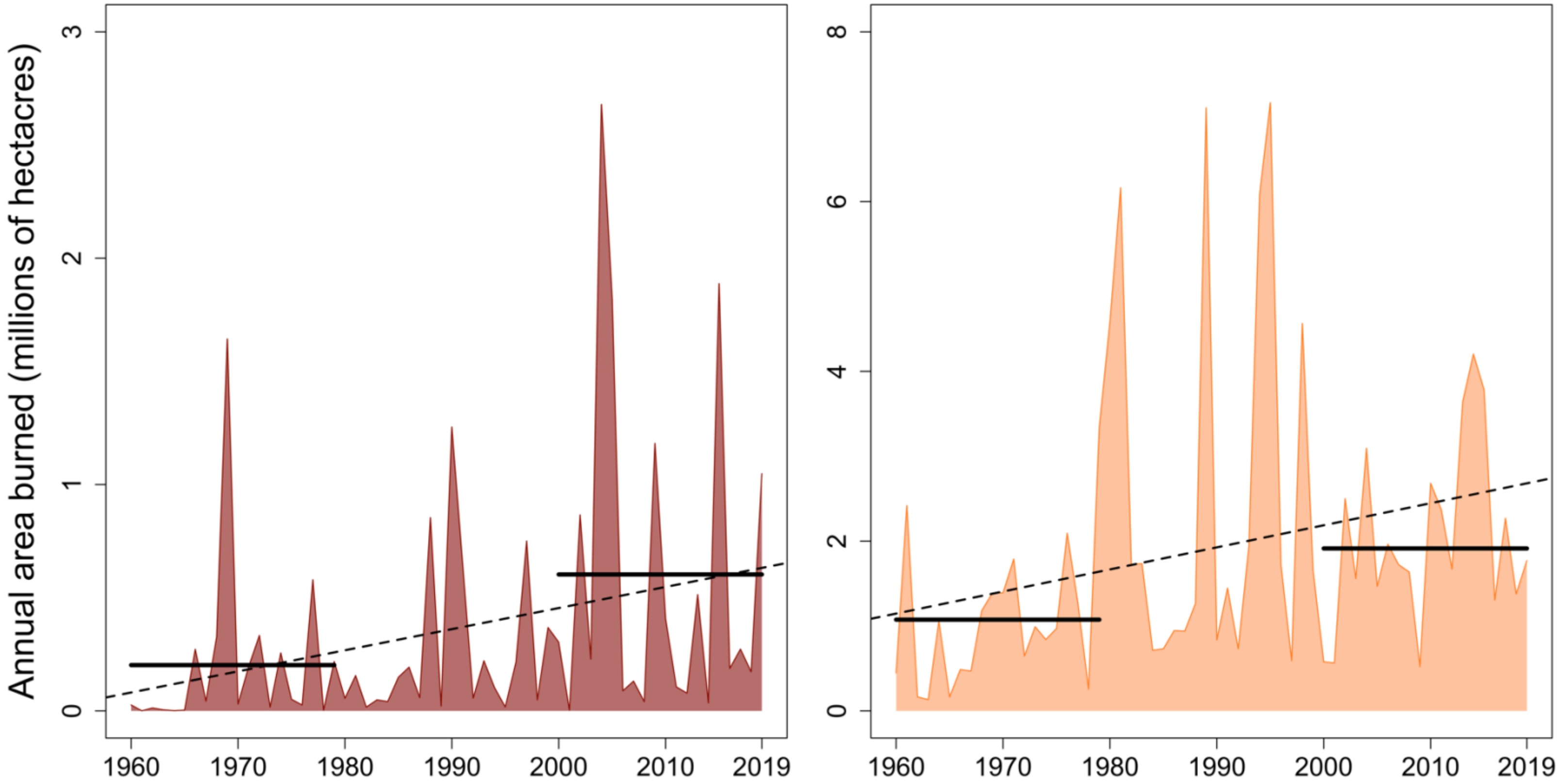

Figure S4: Annual burned area over time (dashed line) since 1960 in Alaska (panel A) and Canada (panel B). Black segments represent annual average annual burned area between 1960-1979 and 2000-2019, a change of 163% (Alaska) and 100% (Canada). Annual burned area in Alaska has been quality-controlled and adjusted using previously published discrepancies (*68*). Note the difference in scale of y-axis between panels.

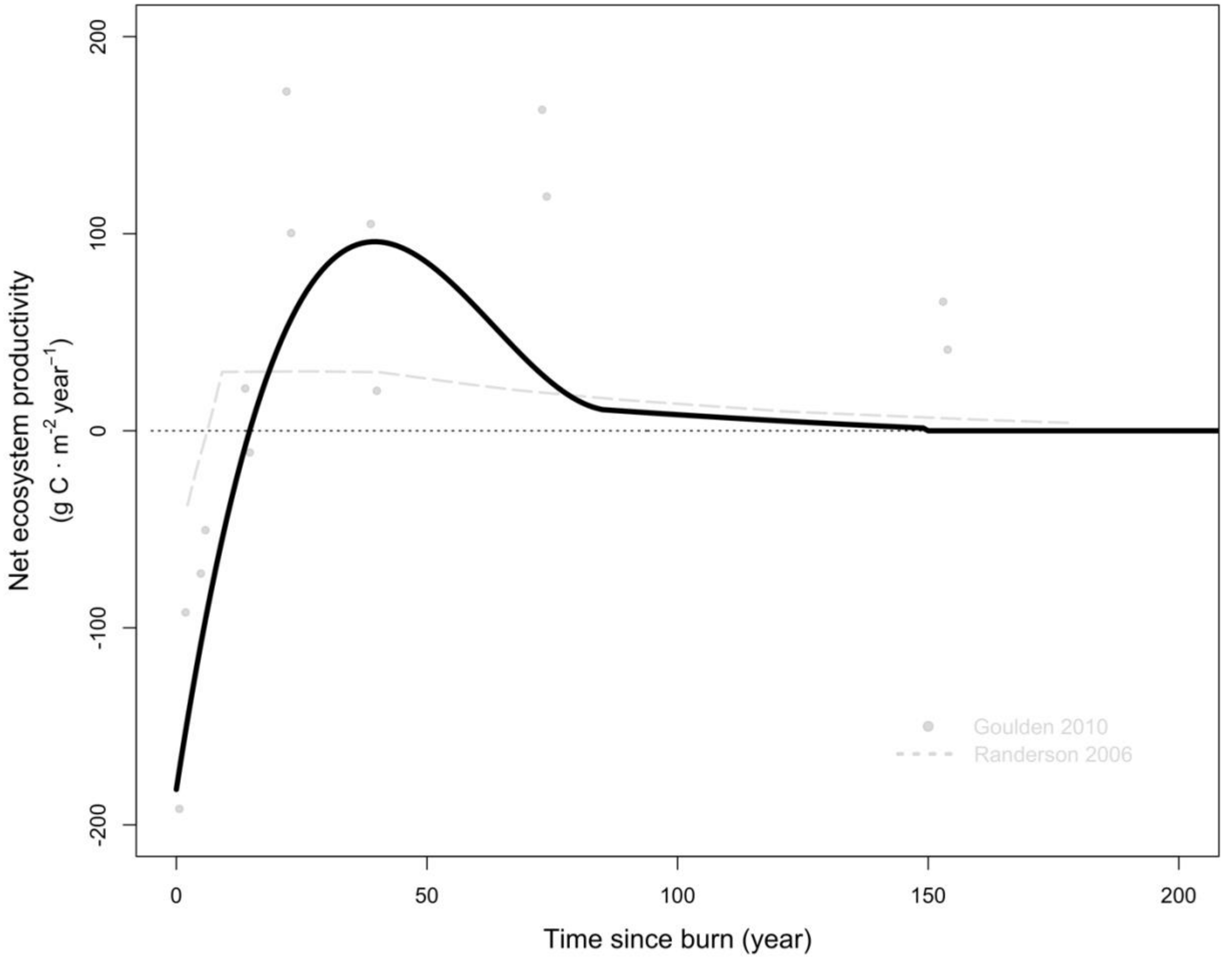

Fig S5: Regeneration curve showing net ecosystem productivity following a fire (*35,66*)

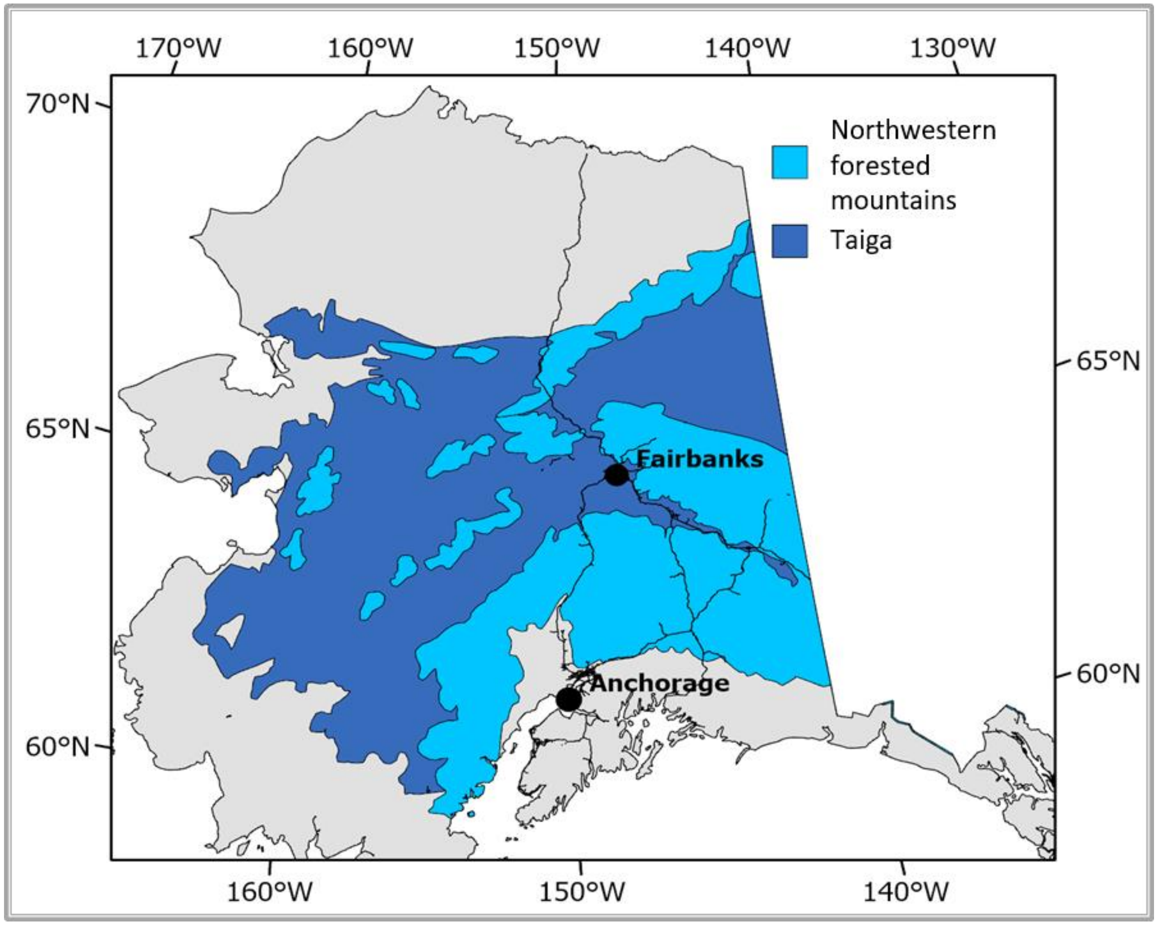

Figure S6: Study region of Alaska's boreal forests characterized by EPA-defined Level I Ecoregions, including taiga and northwestern forested mountains (*67*)

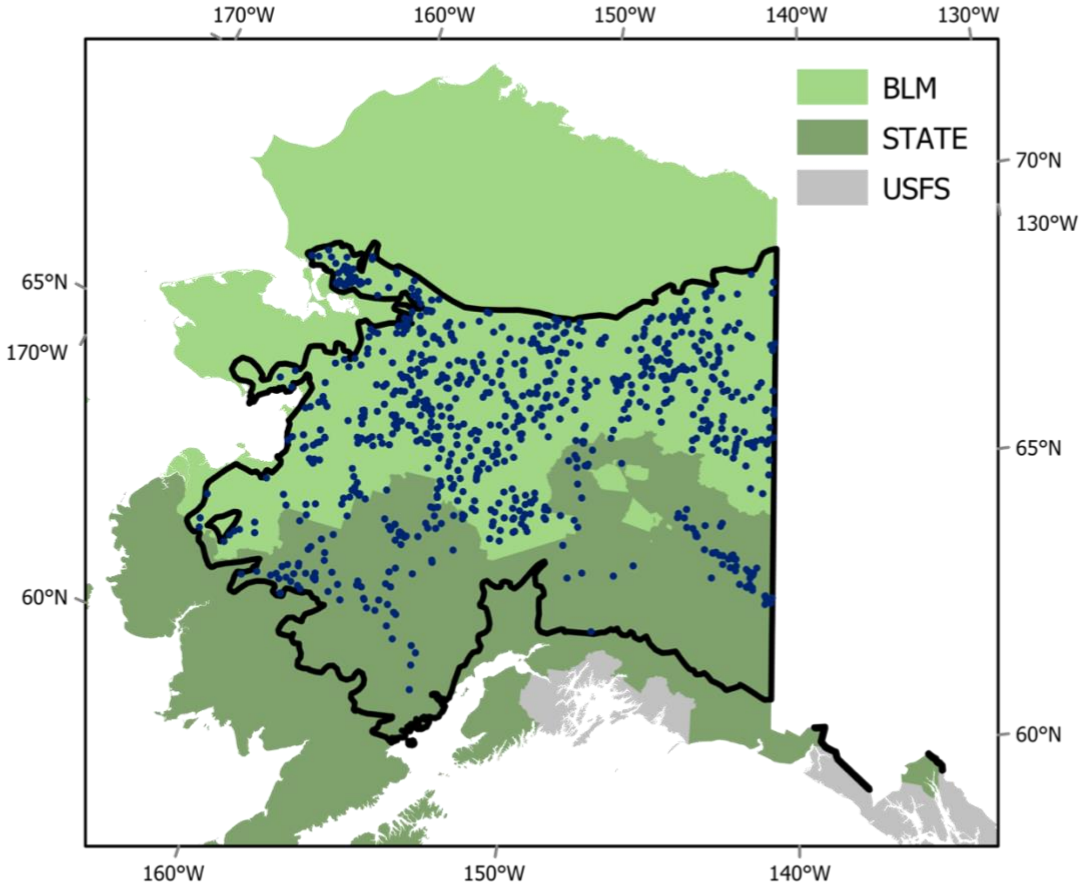

Figure S7: Location of fires used in economic analysis as distributed across protection jurisdictions of Bureau of Land Management, State of Alaska and United States Forest Service. These jurisdictions represent the agency in charge of fire protection efforts. Black border indicates boreal zone used for analysis.

Table S1: Summary table of Alaska, Canada, and North American boreal fire annual and cumulative emissions (net & gross) for historical, modern, and projected future periods. Methods on emissions projections available in main text.

	**Alaska**	**Canada**	**Boreal North America (Alaska + Canada)**
**Average annual net emissions (Gt CO_2)**			
Historical (1960-1979)	0.001	0.006	0.007
Modern (2000-2019)	0.054	0.111	0.166
Projected average (2021-2050)	0.084	0.137	0.221
Projected lower (2021-2050)	0.022	0.023	0.045
Projected upper (2021-2050)	0.146	0.251	0.398
**Average annual gross emissions (Gt CO_2)**			
Historical (1960-1979)	0.021	0.110	0.131
Modern (2000-2019)	0.062	0.208	0.270
Projected average (2021-2050)	0.099	0.297	0.396
Projected lower (2021-2050)	0.050	0.206	0.256
Projected upper (2021-2050)	0.148	0.387	0.535
**Cumulative net emissions (Gt CO_2)**			
Historical (1960-1979)	0.023	0.123	0.146
Modern (2000-2019)	1.085	2.227	3.312
Projected average (2021-2050)	2.525	4.108	6.633
Projected lower (2021-2050)	0.656	0.680	1.336
Projected upper (2021-2050)	4.394	7.537	11.931
**Cumulative gross emissions (Gt CO_2)**			
Historical (1960-1979)	0.414	2.202	2.616
Modern (2000-2019)	1.235	4.166	5.401
Projected average (2021-2050)	2.969	8.898	11.867
Projected lower (2021-2050)	1.498	6.192	7.690
Projected upper (2021-2050)	4.440	11.604	16.044

Table S2: First stage of Two Stage Least Squares IV econometrics approach: Regression of instrumented variable, cost on instruments for different fire management zones

FMZ	lntotalcost
modified	2.3137
	(0.3413)**
full	2.8448
	(0.3201)**
critical	1.9782
	(0.7519)**
R^2	0.62
N	832

* *p*<0.05; ** *p*<0.01 Controls in preferred models include mean and maximum values of FFMC, ISI, BUI, FWI, DSR (definitions provided in Table S8) temperature, relative humidity, snow depth, and windspeed, mean DMC, maximum DC, elevation, white and black spruce coverage, deciduous coverage, grassland coverage, "burnable" coverage, latitude and longitude, and month and year fixed effects.

Table S3: Main econometric results (Second stage of 2SLS IV) where the positive coefficient in the naïve OLS regression captures the reverse causality of increasing fire size driving costs up. The IV regression gives the preferred specification, and the negative coefficient captures the causal relationship of expenditures bringing down burned area.

	OLS	IV
lntotalcost	0.1621	-0.2063
	(0.0305)**	(0.0906)*
R^2	0.65	0.58
N	857	832

* *p*<0.05; ** *p*<0.01 Controls in preferred models include mean and maximum values of FFMC, ISI, BUI, FWI, DSR, temperature, relative humidity, snow depth, and windspeed, mean DMC, maximum DC, elevation, white and black spruce coverage, deciduous coverage, grassland coverage, "burnable" coverage, latitude and longitude, and month and year fixed effects.

Table S4: Alternate specifications for IV model where *IV column* applies the preferred specification to BLM costs rather than total costs, the *lightning only column* excludes human-caused fires in case they are responded to differently. Columns 3 and 4 (log(Cost + 0.01) and IHS cost) use different transformations to deal with the $0 cost fires in case the particular transformation used in the preferred specification (natural log of cost + 1) is affecting results. None of the alternate specifications yield statistically distinguishable results from the preferred specification.

	IV	Lightning Only	log(Cost + 0.01)	IHS Cost
lnblmcost	-0.2487<br>(0.1024)*			
lntotalcost		-0.2046<br>(0.0934)*	-0.1799<br>(0.0786)*	
ihstotalcost				-0.2017<br>(0.0885)*
R^2	0.57	0.54	0.58	0.58
N	857	762	832	832

* *p*<0.05; ** *p*<0.01 Controls in preferred models include mean and maximum values of FFMC, ISI, BUI, FWI, DSR, temperature, relative humidity, snow depth, and windspeed, mean DMC, maximum DC, elevation, white and black spruce coverage, deciduous coverage, grassland coverage, "burnable" coverage, latitude and longitude, and month and year fixed effects.

Table S5: Estimates of CO_2 emissions from fire suppression activities in AK boreal forests. Emissions were calculated using separate coefficients for jet fuel (2.53 kg CO_2/liter) and gasoline (2.35 kg CO_2/liter). On average, emissions from suppression efforts represent, on average, 0.57% of total annual net wildfire emissions.

Year	Total area burned (hectares)	Net annual wildfire emissions (Tg CO_2)	Total spending	Estimated fuel expenditures	Jet fuel price ($/liter)	Gasoline price ($/liter)	Esstimate jet fuel purchased (liters)	Estimate gasoline purchased (liters)	Jet fuel emissions (kg CO_2)	Gasoline emissions (kg CO_2)	Combined emissions (Tg CO_2)	% of net wildfire emissions
2009	1,187,636	46.41	$94,298,237	$23,574,559	$0.45	$0.81	26,062,913	14,629,412	36,985,005	34,357,021	0.07	0.15
2010	455,457	26.27	$88,739,600	$22,184,900	$0.53	$0.86	20,797,172	12,919,843	32,662,999	30,342,117	0.06	0.24
2011	118,584	5.92	$85,779,755	$21,444,939	$0.83	$1.11	12,963,578	9,664,039	24,431,912	22,695,893	0.05	0.80
2012	116,104	2.52	$40,765,749	$10,191,437	$0.76	$1.03	6,669,915	4,945,998	12,504,110	11,615,625	0.02	0.96
2013	532,702	36.37	$94,528,765	$23,632,191	$0.76	$1.06	15,455,697	11,182,197	28,270,009	26,261,273	0.05	0.15
2014	94,639	2.57	$74,809,624	$18,702,406	$0.74	$1.04	12,565,905	8,984,303	22,713,455	21,099,542	0.04	1.70
2015	2,068,605	152.83	$188,234,826	$47,058,707	$0.41	$0.80	57,500,511	29,298,780	74,071,023	68,807,879	0.14	0.09
2019	1,046,402	54.92	$303,900,000	$75,975,000	$0.51	$0.73	75,169,017	51,912,755	131,242,013	121,916,561	0.25	0.46

Table S6: Remaining CO_2 budgets (Gt CO_2), likelihood of limiting temperatures, and the proportion of remaining net emissions that North American (NA) boreal forests represent. Data on initial carbon budgets and likelihoods from 2021 AR6 WG1 report (*79*).

Target temperature increase limit (°C)	Remaining budget (as of Jan 1 2020)	% chance of limiting increase	% NA boreal wildfires (lower estimate)	% NA boreal wildfires (upper estimate)	Average % NA boreal wildfires
1.5	650	33	0.21%	1.84%	1.02%
	500	50	0.27%	2.39%	1.33%
	**400**	**67**	0.33%	2.98%	**1.66%**
2.0	1700	33	0.08%	0.70%	0.39%
	1350	50	0.10%	0.88%	0.49%
	1150	67	0.12%	1.04%	0.58%

Table S7: Papers evaluated to assess projected CO_2 emissions through 2050 from Alaskan and Canadian boreal wildfires.

Paper	Region	Projected increase (%)	Projection Year	% increase at 2050	Global circulation model (models in ensemble)	Modeling technique	Scenario
Amiro et al. *(58)*	Canada	34	2050	34	CGCM1	FBP	2 x CO_2, 3 x CO_2
Flannigan et al. *(78)*	Canada	96	2100	36	CGCM1, HadCM3G	Linear forward stepwise regression	SRES A1B
Flannigan and Wagner 1991 *(74)*	Canada	46	2050	46	GCMS (3)	Linear regression	2 x CO_2
Boulanger et al. *(77)*	Canada	140	2100	52.5	CanESM2, HadGEM, MIROC	MARS, GLM, random forest, GBM	RCPs 2.6, 4.5, 8.5
Yue et al. *(60)*	Canada	71	2050	71	CMIP3 (13)	USFS CONSUME	RCPs 4.5, 6.0
Boulanger et al. *(61)*	Canada	270	2100	101.25	CGCM3-T47, CRCM 4.2.0	MARS	SRES A2
Gauthier et al. *(76)*	Canada	400	2070	150	CGCM3-T47, CRCM 4.2.0	MARS	RCPs 4.5, 8.5
Hope et al. *(62)*	Canada	313	2055	313	CanESM2, CESM1CAM5, HadGEM2-ES, MIROC-ESM	Linear regression	RCPs 2.6, 8.5
Bachelet et al. *(75)*	Alaska	24	2099	9.73	AOGCM, CGCM1, HADCM2SUL	MC1 (DGVM)	IS92
Genet et al. *(34)*	Alaska	33	2100	14.67	CCMA	DOS-TEM, ALFRESCO	SRES A1B
Genet et al. *(58)*	Alaska	44	2100	22	CGCM3.1 (3)	DOS-TEM, MDM-TEM, ALFRESCO	SRES B1, A1B, A2
Mekonnen et al. *(37)*	Alaska	71	2100	26.63	CMIP5 GCMs (15)	ecosys	RCPs 8.5
Euskirchen et al. *(63)*	Alaska	90	2100	43.61	Hadley CM3, PCM	TEM, ALFRESCO	SRES B2, A2
Pastick et al. *(21)*	Alaska	150	2100	50	ECHAM, CCCMA	DOS-TEM, ALFRESCO	SRES B1, A1B, A2
Veraverbeke et al. *(8)*	Alaska	55	2050	55	CMIP5 GCMs (5)	Multiple regression	RCP 8.5
Melvin et al. *(53)*	Alaska	87	2050	87	CMIP5 GCMs (5)	ALFRESCO	RCPs 4.5, 8.5
Young et al. *(64)*	Alaska	222	2100	98.67	CMIP5 (5)	Boosted regression tree	RCP 6
Balshi et al. *(20)*	Alaska	350	2100	163.83	Canadian CGCM2	MARS	SRES A2, B2
Balshi et al. *(28)*	Alaska	360	2100	174.43	CGCM2	TEM, MARS	SRES A2, B2
Kitzberger et al. *(22)*	Alaska	150	2039	200	GCMs (21)	Multiple regression, SEM	SRES A1B
Yue et al. *(60)*	Alaska	265	2050	265	CMIP3 (13)	USFS CONSUME	RCP 4.5, 6.0

Table S8: Categorization of fire cause for fires from 2000-2018 in Alaska's boreal forests

**Modeled category**	**Total Number of Fires**	**Fire history general cause**
Lightning	2356	Lightning - WFU
Human	852	Other Human Cause
		Children
		Smoking
		Debris Burning
		Debris/Open Burning
		Firearms/Weapons
		Incendiary
		Campire
		Camping
Undetermined	203	Cause and Origin Not Identified
		Miscellaneous
		Natural Out
		NA

Table S9: Unburnable vegetation categories in LANDFIRE. Categorization taken from 2016 BLM Alaska Fire Service Wildfire Workload Analysis.

Unburnable Vegetation - LANDFIRE	
**EVT Name**	**EVT Code**
Barren (non-vegetated)	0031
Snow-Ice	0012
Open water	0011
Boreal aquatic beds	2740
Polar tidal marches and aquatic beds	2741
Temperatre Pacific tidal marshes, aquatic beds, and intertidal flats	2742
Alaska arctic non-acidic sparse tundra	2686
Arctic sparsely vegetated	2792
Boreal sparsely vegetated	2793
Pacific maritime sparsely vegetated	2794
Aleutian Sparsely vegetated	2791
Aleutian sparse health and fell-field	2730
Alaska Pacific maritime alpine sparse shrub and fell-field	2674

Table S10: Climate and weather variables initially considered for our random forest model. We calculated daily maximum and mean of each index over the duration of each individual fire. Bolded variables indicate inclusion in the final model. Definitions are adapted from the National Wildfire Coordinating Group.

Abbreviation	Index	Definition	Measurement
BUI	Buildup index	estimates heat release from fuels, approximates burn severity	unconstrained, unitless scale
DC	Drought code	estimates moisture conditions into organic soil by using moisture conditions from 53 days prior	unitless scale up to 1000
**DMC**	**Duff moisture code**	indicates moisture content of decomposed organic material beneath the litter layer	unconstrained, unitless scale
DSR	Daily severity rating	rates the difficulty of controlling fires	unconstrained, unitless scale
**FFMC**	**Fine fuel moisture code**	indicates moisture content of litter layer	unitless scale of 1-101
FWI	Fire weather index	estimates general fire intensity	unconstrained, unitless scale
ISI	Initial spread index	approximates spread potential	unconstrained, unitless scale
**t**	**Temperature**	temperature	°C
VPD	Vapor pressue deficit	measures the difference between current air moisture and air moisture saturation potential	kiloPascals (kPa)
wdSpd	Wind speed	wind speed	miles per hour

Supplementary Methods

A. Literature search

For Alaska, our Web of Science search with the terms [(fire OR wildfire) AND (Alaska OR AK) AND (project* OR model* predict*)] yielded 464 papers. For Canada, our search with [(fire OR wildfire) AND (Canada OR CA) AND (project* OR model* predict*) AND (burned area OR area burned OR area)] yielded 1394 results. We initially excluded duplicates, papers on unrelated topics or regions, and those that projected burned area for subsets of the entire state or country. We included papers that projected burned area, not fire weather, fire occurrence, length of fire seasons or other adjacent metrics. We further only included papers that projected over specific periods of time, not over unspecified decades (i.e., no provided start/end date).

Assessing the relationship between fire management and burned area in Alaska

B. Fire history data

We used both the point and polygon data in our analysis. For all variables except vegetation, we extracted information based on the point dataset that includes smaller fires to better capture the influence of management. Fires of all sizes are present in the point dataset for a total of approximately 31,000 fires. In contrast, only fires larger than 100 hectares are included in the polygon data between 1987-2015. After 2016, fires over 10 hectares are included. The polygon dataset contains approximately 3,700 fires. These datasets also include 'False Alarm' fires that were reported but did not burn. To ensure our model included only true wildfires, we removed these false alarm fires as well as prescribed burns. Further, due to their importance in calculating fire weather indices, we removed fires for which we could not calculate a burn duration (i.e., those that lacked either a discovery date or out date).

C. Fire management zones (FMZs)

Fires in the Critical protection zone receive the swiftest and most aggressive suppression, and threaten human life, inhabited structures, and landmarks. On the opposite end of the spectrum, fires in the Limited protection zone are typically monitored without intervention, as they are in regions far removed from human development where the values on land, as currently defined, are not sufficient to justify the expense of suppression. An intermediate suppression zone, fires in Full area are considered second priority, and threaten uninhabited private property, natural resources, and historical and cultural sites. Modified areas are considered in Full protection until a conversion date, after which they become part of a third priority designation beneath both Critical and Full.

While non-standard responses to fires are possible (i.e., when action on an individual fire deviates from the management plan), these designations largely determine the initial response and resources allocated to any given fire. FMZs can be adjusted by jurisdictional agencies each year. To account for these annual changes, we extracted management zone from historic FMZ layers that corresponded to the appropriate burn year. For fires in Modified areas, we used annual conversion dates and date of discovery to categorize fires as either Full (discovered before the conversion date) or Modified protection (discovered after the conversion date). Detailed information about the history, purpose, and goals of fire management zones can be found in the most 2016 update of the Alaska Interagency Wildfire Management Plan.

D. Random forest variable selection and parameterization

For our random forest model that we used to assess the relationship between fire management zone and fire size, we started with 29 predictor variables and selected 10 to use in our subsequent model. To do so, we used a correlation matrix to identify pair-wise correlations. We removed 10 variables to reduce pair-wise correlation, such that no two variables had a coefficient less than 0.8. using Pearson's correlation. Next, we used recursive feature elimination to determine which variables had the highest predictive power, and thus which correlated variables to include in our model.

E. Instrumental variable approach

Burned area may affect the cost of a fire because, other things held constant, the larger a fire grows, the more it may cost to manage. This effect is evident in the positive coefficient on cost in Column 1 of Table S7. To account for this, we used a two-stage instrumental variables (IV) approach. FMZ could also potentially influence fire size directly if fires differ non-randomly by zone, either because they are started in different ways, or because discovery takes longer or suppression costs are less efficient further from population centers. To address the first concern, we control for fire cause (human or lightning). Although we are not overly concerned about the second potential issue, in particular because remote fire detection relies on satellite data that is updated at minimum twice daily during our sample period, this remains a caveat. Furthermore, even if the time from ignition to discovery is slightly correlated with FMZ, it seems unlikely to significantly affect outcomes, since fires in the limited zone which may take longer to discover and are not generally attacked upon discovery, except in high fire risk situations.

F. Variable selection using LASSO

The LASSO works by minimizing the sum of squared residuals plus a penalty term based on the sum of all estimated coefficients. This method incentivizes LASSO to set some coefficients to zero. We can thus use the LASSO for model selection by observing to which control variables the LASSO assigns coefficients of zero, and then running the desired 2SLS IV regressions excluding those variables. However, there may be controls that we do not wish to exclude, whether or not they are individually significant. For this paper, these necessary controls include year, month, and ecoregion dummies, and latitude and longitude. To run the LASSO with these controls already included, we regress the outcome variable(s) on the desired included controls only, and then use the residuals as the new outcome variable for the LASSO. We also use a double debiased approach, run the LASSO on the outcome variable and candidate control variables, excluding the treatment, and separately run the LASSO on the treatment and the candidate controls. Controls with non-zero coefficients in either result are included in post-LASSO regressions. Running both specifications helps avoid introducing omitted variables bias. The resulting coefficients are slightly smaller than in the stepwise variable elimination approach, but are not statistically distinct.

G. Estimating future fire suppression costs

Using annual aggregated cost data, we multiplied the cost of averting one ton of CO_2 (~$12/ton) by the difference between each year's (2021-2050) projected emissions (upper, lower & mean) and average historic emissions as described in the main text methods section titled *CO_2 emissions*. For each year, this allowed us to quantify the range of costs required to reduce emissions to historic levels. We further calculated the average annual cost of an Alaskan fire season using the eight years for which we had data on total suppression expenditures.

H. Road analysis

To account for the challenges of fighting wildfire in remote areas, we compared the cost per ton of averting emissions for fires that ignited near roads and those in roadless areas. We combined roads from two publicly available datasets (Alaska Roads 1:1,000,000, 1995; Alaska Roads 1:63,360, 2006) and included roads that were functional or under construction (1: 1mil) as well as the full dataset of roads from the 1:1:63,360. Our final dataset included roughly 11,545 km of roads within our study area. We then created a 10 km buffer around all roads. We considered fires within this buffer as "roaded," and those that lay outside as "roadless."

Datasets

A) Data table S1: Aggregated fire suppression expenditures expressed as USD 2020. These data were obtained through Kent Slaughter, Deputy State Director, Fire and Aviation with the Alaska Fire Service under the Bureau of Land Management.

Year	State of Alaska Division of Forestry General Fund	State of Alaska Division of Forestry Federal Fund	State of Alaska Division of Forestry Suppression Total	Bureau of Land Management Alaska Fire Suppression Costs	Combined TOTAL
**2009**	$52,152,511.97	$17,902,103.37	$70,054,615.35	$42,714,126.00	$112,768,741.35
**2010**	$46,467,284.08	$11,592,735.00	$58,060,019.07	$46,842,257.26	$104,902,276.34
**2011**	$57,804,663.85	$19,300,836.50	$77,105,500.35	$22,222,027.03	$99,327,527.37
**2012**	$15,101,396.12	$19,694,642.76	$34,796,038.88	$11,519,016.99	$46,315,055.87
**2013**	$48,412,871.48	$19,846,385.42	$68,259,256.89	$37,287,664.51	$105,546,921.41
**2014**	$32,218,060.86	$26,126,183.15	$58,344,244.00	$23,668,979.64	$82,013,223.64
**2015**	$81,448,371.80	$55,524,189.79	$136,972,561.58	$67,434,653.93	$204,407,215.51
**2019**	NA	NA	$230,640,102.57	$79,949,838.10	$310,589,940.67

REFERENCES AND NOTES

1. J. Rogelj, D. Shindell, K. Jiang, S. Fifita, P. Forster, V. Ginzburg, C. Handa, H. Kheshgi, S. Kobayashi, E. Kriegler, L. Mundaca, R. Séférian, M. V. Vilarino, K. Calvin, J. C. de Oliveira de Portugal Pereira, O. Edelenbosch, J. Emmerling, S. Fuss, T. Gasser, N. Gillett, C. He, E. Hertwich, L. Höglund-Isaksson, D. Huppmann, G. Luderer, A. Markandya, M. Meinshausen, D. McCollum, R. Millar, A. Popp, P. Purohit, K. Riahi, A. Ribes, H. Saunders, C. Schädel, C. Smith, P. Smith, E. Trutnevyte, Y. Xu, W. Zhou, K. Zickfeld, Mitigation pathways compatible with 1.5°C in the context of sustainable development, in *Global Warming of 1.5 °C* (Intergovernmental Panel on Climate Change, Geneva, 2018), pp. 93–174.

2. D. van Wees, G. Werf, J. Randerson, N. Andela, Y. Chen, D. Morton, The role of fire in global forest loss dynamics. *Glob. Chang. Biol.* **27**, 2377–2391 (2021).

3. E. S. Kasischke, M. R. Turetsky, Recent changes in the fire regime across the North American boreal region—Spatial and temporal patterns of burning across Canada and Alaska. *Geophys. Res. Lett.* **33**, L09703 (2006).

4. R. Kelly, M. L. Chipman, P. E. Higuera, I. Stefanova, L. B. Brubaker, F. S. Hu, Recent burning of boreal forests exceeds fire regime limits of the past 10,000 years. *Proc. Natl. Acad. Sci.* **110**, 13055–13060 (2013).

5. C. J. A. Bradshaw, I. G. Warkentin, Global estimates of boreal forest carbon stocks and flux. *Global Planet. Change* **128**, 24–30 (2015).

6. Y. Pan, R. A. Birdsey, J. Fang, R. Houghton, P. E. Kauppi, W. A. Kurz, O. L. Phillips, A. Shvidenko, S. L. Lewis, J. G. Canadell, P. Ciais, R. B. Jackson, S. W. Pacala, A. D. McGuire, S. Piao, A. Rautiainen, S. Sitch, D. Hayes, A large and persistent carbon sink in the world's forests. *Science* **333**, 988–993 (2011).

7. M. R. Turetsky, E. S. Kane, J. W. Harden, R. D. Ottmar, K. L. Manies, E. Hoy, E. S. Kasischke, Recent acceleration of biomass burning and carbon losses in Alaskan forests and peatlands. *Nat. Geosci.* **4**, 27–31 (2011).

8. S. Veraverbeke, B. M. Rogers, M. L. Goulden, R. R. Jandt, C. E. Miller, E. B. Wiggins, J. T. Randerson, Lightning as a major driver of recent large fire years in North American boreal forests. *Nat. Clim. Chang.* **7**, 529–534 (2017).

9. X. J. Walker, J. L. Baltzer, S. G. Cumming, N. J. Day, C. Ebert, S. Goetz, J. F. Johnstone, S. Potter, B. M. Rogers, E. A. G. Schuur, M. R. Turetsky, M. C. Mack, Increasing wildfires threaten historic carbon sink of boreal forest soils. *Nature* **572**, 520–523 (2019).

10. H. T. Lewis, T. A. Ferguson, Yards, corridors, and mosaics: How to burn a boreal forest. *Hum. Ecol.* **16**, 57–77 (1988).

11. K. M. Hoffman, E. L. Davis, S. B. Wickham, K. Schang, A. Johnson, T. Larking, P. N. Lauriault, N. Q. Le, E. Swerdfager, A. J. Trant, Conservation of Earth's biodiversity is embedded in Indigenous fire stewardship. *Proc. Natl. Acad. Sci. U.S.A.* **118**, e2105073118 (2021).

12. B. M. Rogers, A. J. Soja, M. L. Goulden, J. T. Randerson, Influence of tree species on continental differences in boreal fires and climate feedbacks. *Nat. Geosci.* **8**, 228–234 (2015).

13. C. C. Hanes, X. Wang, P. Jain, M.-A. Parisien, J. M. Little, M. D. Flannigan, Fire-regime changes in Canada over the last half century. *Can. J. For. Res.* **49**, 256–269 (2019).

14. C. Tymstra, B. J. Stocks, X. Cai, M. D. Flannigan, Wildfire management in Canada: Review, challenges and opportunities. *Prog. Disaster Sci.* **5**, 100045 (2020).

15. M. P. Girardin, A. A. Ali, C. Carcaillet, S. Gauthier, C. Hély, H. Le Goff, A. Terrier, Y. Bergeron, Fire in managed forests of eastern Canada: Risks and options. *For. Ecol. Manage.* **294**, 238–249 (2013).

16. R. V. Bekryaev, I. V. Polyakov, V. A. Alexeev, Role of polar amplification in long-term surface air temperature variations and modern arctic warming. *J. Climate* **23**, 3888–3906 (2010).

17. V. Masson-Delmotte, M. Kageyama, P. Braconnot, S. Charbit, G. Krinner, C. Ritz, E. Guilyardi, J. Jouzel, A. Abe-Ouchi, M. Crucifix, R. M. Gladstone, C. D. Hewitt, A. Kitoh, A. N. LeGrande, O. Marti, U. Merkel, T. Motoi, R. Ohgaito, B. Otto-Bliesner, W. R. Peltier, I. Ross, P. J. Valdes, G.

Vettoretti, S. L. Weber, F. Wolk, Y. Yu, Past and future polar amplification of climate change: Climate model intercomparisons and ice-core constraints. *Climate Dynam.* **26**, 513–529 (2006).

18. A. Krause, S. Kloster, S. Wilkenskjeld, H. Paeth, The sensitivity of global wildfires to simulated past, present, and future lightning frequency. *J. Geophys. Res. Biogeo.* **119**, 312–322 (2014).

19. D. M. Romps, J. T. Seeley, D. Vollaro, J. Molinari, Projected increase in lightning strikes in the United States due to global warming. *Science* **346**, 851–854 (2014).

20. M. S. Balshi, A. D. Mcguire, P. Duffy, M. Flannigan, D. W. Kicklighter, J. Melillo, Vulnerability of carbon storage in North American boreal forests to wildfires during the 21st century. *Glob. Chang. Biol.* **15**, 1491–1510 (2009).

21. N. J. Pastick, P. Duffy, H. Genet, T. S. Rupp, B. K. Wylie, K. D. Johnson, M. T. Jorgenson, N. Bliss, A. D. McGuire, E. E. Jafarov, J. F. Knight, Historical and projected trends in landscape drivers affecting carbon dynamics in Alaska. *Ecol. Appl.* **27**, 1383–1402 (2017).

22. T. Kitzberger, D. A. Falk, A. L. Westerling, T. W. Swetnam, Direct and indirect climate controls predict heterogeneous early-mid 21st century wildfire burned area across western and boreal North America. *PLOS ONE* **12**, e0188486 (2017).

23. S. Veraverbeke, B. M. Rogers, J. T. Randerson, Daily burned area and carbon emissions from boreal fires in Alaska. *Biogeosciences* **12**, 3579–3601 (2015).

24. B. M. Rogers, S. Veraverbeke, G. Azzari, C. I. Czimczik, S. R. Holden, G. O. Mouteva, F. Sedano, K. K. Treseder, J. T. Randerson, Quantifying fire-wide carbon emissions in interior Alaska using field measurements and Landsat imagery. *J. Geophys. Res. Biogeosci.* **119**, 1608–1629 (2014).

25. F. Oris, H. Asselin, A. A. Ali, W. Finsinger, Y. Bergeron, Effect of increased fire activity on global warming in the boreal forest. *Environ. Rev.* **22**, 206–219 (2014).

26. S. Akagi, R. Yokelson, C. Wiedinmyer, M. Alvarado, J. Reid, T. Karl, J. Crounse, P. Wennberg, Emission factors for open and domestic biomass burning for use in atmospheric models. *Atmos. Chem. Phys.* 4039–4072 (2011).

27. D. R. N. Brown, M. T. Jorgenson, T. A. Douglas, V. E. Romanovsky, K. Kielland, C. Hiemstra, E. S. Euskirchen, R. W. Ruess, Interactive effects of wildfire and climate on permafrost degradation in Alaskan lowland forests. *J. Geophys. Res. Biogeo.* **120**, 1619–1637 (2015).

28. M. S. Balshi, A. D. McGuirez, P. Duffy, M. Flannigan, J. Walsh, J. Melillo, Assessing the response of area burned to changing climate in western boreal North America using a Multivariate Adaptive Regression Splines (MARS) approach. *Glob. Chang. Biol.* **15**, 578–600 (2009).

29. M. P. Calef, A. Varvak, A. D. McGuire, F. S. Chapin, K. B. Reinhold, Recent changes in annual area burned in interior Alaska: The impact of fire management. *Earth Interact.* **19**, 1–17 (2015).

30. C. Strobl, A.-L. Boulesteix, T. Kneib, T. Augustin, A. Zeileis, Conditional variable importance for random forests. *BMC Bioinformatics* **9**, 307 (2008).

31. S. R. Coffield, C. A. Graff, Y. Chen, P. Smyth, E. Foufoula-Georgiou, J. T. Randerson, Machine learning to predict final fire size at the time of ignition. *Int. J. Wildland Fire* **28**, 861–873 (2019).

32. D. R. N. Brown, M. T. Jorgenson, K. Kielland, D. L. Verbyla, A. Prakash, J. C. Koch, Landscape effects of wildfire on permafrost distribution in interior Alaska derived from remote sensing. *Remote Sens.* **8**, 654 (2016).

33. S. M. Natali, J. P. Holdren, B. M. Rogers, R. Treharne, P. B. Duffy, R. Pomerance, E. MacDonald, Permafrost carbon feedbacks threaten global climate goals. *Proc. Natl. Acad. Sci. U.S.A.* **118**, e2100163118 (2021).

34. H. Genet, A. D. McGuire, K. Barrett, A. Breen, E. S. Euskirchen, J. F. Johnstone, E. S. Kasischke, A. M. Melvin, A. Bennett, M. C. Mack, T. S. Rupp, A. E. G. Schuur, M. R. Turetsky, F. Yuan, Modeling the effects of fire severity and climate warming on active layer thickness and soil carbon storage of black spruce forests across the landscape in interior Alaska. *Environ. Res. Lett.* **8**, 045016 (2013).

35. J. T. Randerson, H. Liu, M. G. Flanner, S. D. Chambers, Y. Jin, P. G. Hess, G. Pfister, M. C. Mack, K. K. Treseder, L. R. Welp, F. S. Chapin, J. W. Harden, M. L. Goulden, E. Lyons, J. C. Neff, E. A.

G. Schuur, C. S. Zender, The impact of boreal forest fire on climate warming. *Science* **314**, 1130–1132 (2006).

36. D. S. Ward, S. Kloster, N. M. Mahowald, B. M. Rogers, J. T. Randerson, P. G. Hess, The changing radiative forcing of fires: Global model estimates for past, present and future. *Atmos. Chem. Phys.* **12**, 10857–10886 (2012).

37. Z. A. Mekonnen, W. J. Riley, J. T. Randerson, R. F. Grant, B. M. Rogers, Expansion of high-latitude deciduous forests driven by interactions between climate warming and fire. *Nat. Plants* **5**, 952–958 (2019).

38. J. L. Baltzer, N. J. Day, X. J. Walker, D. Greene, M. C. Mack, H. D. Alexander, D. Arseneault, J. Barnes, Y. Bergeron, Y. Boucher, L. Bourgeau-Chavez, C. D. Brown, S. Carrière, B. K. Howard, S. Gauthier, M.-A. Parisien, K. A. Reid, B. M. Rogers, C. Roland, L. Sirois, S. Stehn, D. K. Thompson, M. R. Turetsky, S. Veraverbeke, E. Whitman, J. Yang, J. F. Johnstone, Increasing fire and the decline of fire adapted black spruce in the boreal forest. *Proc. Natl. Acad. Sci. U.S.A.* **118**, e2024872118 (2021).

39. J. F. Johnstone, T. N. Hollingsworth, F. S. Chapin III, M. C. Mack, Changes in fire regime break the legacy lock on successional trajectories in Alaskan boreal forest. *Glob. Chang. Biol.* **16**, 1281–1295 (2010).

40. M. T. Jorgenson, V. Romanovsky, J. Harden, Y. Shur, J. O'Donnell, E. A. G. Schuur, M. Kanevskiy, S. Marchenko, Resilience and vulnerability of permafrost to climate change. This article is one of a selection of papers from The Dynamics of Change in Alaska's Boreal Forests: Resilience and Vulnerability in Response to Climate Warming. *Can. J. For. Res.* **40**, 1219–1236 (2010).

41. B. M. Rogers, J. T. Randerson, G. B. Bonan, High-latitude cooling associated with landscape changes from North American boreal forest fires. *Biogeosciences* **10**, 699–718 (2013).

42. M. C. Arienti, S. G. Cumming, S. Boutin, Empirical models of forest fire initial attack success probabilities: The effects of fuels, anthropogenic linear features, fire weather, and management. *Can. J. For. Res.* **36**, 3155–3166 (2006).

43. R. C. Scholten, R. Jandt, E. A. Miller, B. M. Rogers, S. Veraverbeke, Overwintering fires in boreal forests. *Nature* **593**, 399–404 (2021).

44. N. P. Gillett, A. J. Weaver, F. W. Zwiers, M. D. Flannigan, Detecting the effect of climate change on Canadian forest fires. *Geophys. Res. Lett.* **31**, L18211 (2004)

45. E. S. Kasischke, D. L. Verbyla, T. S. Rupp, A. D. McGuire, K. A. Murphy, R. Jandt, J. L. Barnes, E. E. Hoy, P. A. Duffy, M. Calef, M. R. Turetsky, Alaska's changing fire regime — Implications for the vulnerability of its boreal forestsThis article is one of a selection of papers from The Dynamics of Change in Alaska's Boreal Forests: Resilience and Vulnerability in Response to Climate Warming. *Can. J. For. Res.* **40**, 1313–1324 (2010).

46. P. F. Hessburg, C. L. Miller, S. A. Parks, N. A. Povak, A. H. Taylor, P. E. Higuera, S. J. Prichard, M. P. North, B. M. Collins, M. D. Hurteau, A. J. Larson, C. D. Allen, S. L. Stephens, H. Rivera-Huerta, C. S. Stevens-Rumann, L. D. Daniels, Z. Gedalof, R. W. Gray, V. R. Kane, D. J. Churchill, R. K. Hagmann, T. A. Spies, C. A. Cansler, R. T. Belote, T. T. Veblen, M. A. Battaglia, C. Hoffman, C. N. Skinner, H. D. Safford, R. B. Salter, Climate, environment, and disturbance history govern resilience of Western North American forests. *Front. Ecol. Evol.* **7**, 27 (2019).

47. B. M. Collins, R. G. Everett, S. L. Stephens, Impacts of fire exclusion and recent managed fire on forest structure in old growth Sierra Nevada mixed-conifer forests. *Ecosphere*, art51 (2016).

48. R. Astrup, P. Y. Bernier, H. Genet, D. A. Lutz, R. M. Bright, A sensible climate solution for the boreal forest. *Nat. Clim. Chang.* **8**, 11–12 (2018).

49. D. K. Thompson, D. Schroeder, S. L. Wilkinson, Q. Barber, G. Baxter, H. Cameron, R. Hsieh, G. Marshall, B. Moore, R. Refai, C. Rodell, T. Schiks, G. J. Verkaik, J. Zerb, Recent crown thinning in a boreal black spruce forest does not reduce spread rate nor total fuel consumption: Results from an experimental crown fire in Alberta, Canada. *Fire.* **3**, 28 (2020).

50. S. L. Wilkinson, P. A. Moore, D. K. Thompson, B. M. Wotton, S. Hvenegaard, D. Schroeder, J. M. Waddington, The effects of black spruce fuel management on surface fuel condition and peat burn severity in an experimental fire. *Can. J. For. Res.* **48**, 1433–1440 (2018).

51. J. L. Beverly, S. E. R. Leverkus, H. Cameron, D. Schroeder, Stand-level fuel reduction treatments and fire behaviour in canadian boreal conifer forests. *Fire* **3**, 35 (2020).

52. M.-A. Parisien, Q. E. Barber, K. G. Hirsch, C. A. Stockdale, S. Erni, X. Wang, D. Arseneault, S. A. Parks, Fire deficit increases wildfire risk for many communities in the Canadian boreal forest. *Nat. Commun.* **11**, 2121 (2020).

53. A. M. Melvin, J. Murray, B. Boehlert, J. A. Martinich, L. Rennels, T. S. Rupp, Estimating wildfire response costs in Alaska's changing climate. *Clim. Change* **141**, 783–795 (2017).

54. J. C. Liu, G. Pereira, S. A. Uhl, M. A. Bravo, M. L. Bell, A systematic review of the physical health impacts from non-occupational exposure to wildfire smoke. *Environ. Res.* **136**, 120–132 (2015).

55. R. Mathur, Estimating the impact of the 2004 Alaskan forest fires on episodic particulate matter pollution over the eastern United States through assimilation of satellite-derived aerosol optical depths in a regional air quality model. *J. Geophys. Res. Atmos.* **113**, D17302 (2008).

56. S. F. Trainor, M. Calef, D. Natcher, F. S. Chapin III, A. D. McGuire, O. Huntington, P. Duffy, T. Scott Rupp, L. DeWilde, M. Kwart, N. Fresco, A. L. Lovecraft, Vulnerability and adaptation to climate-related fire impacts in rural and urban interior Alaska. *Polar Res.* **28**, 100–118 (2009).

57. J. L. Nelson, E. S. Zavaleta, F. S. Chapin III, Boreal fire effects on subsistence resources in Alaska and adjacent Canada. *Ecosystems* **11**, 156–171 (2008).

58. H. Genet, Y. He, Z. Lyu, A. D. McGuire, Q. Zhuang, J. Clein, D. D'Amore, A. Bennett, A. Breen, F. Biles, E. S. Euskirchen, K. Johnson, T. Kurkowski, S. (Kushch) Schroder, N. Pastick, T. S. Rupp, B. Wylie, Y. Zhang, X. Zhou, Z. Zhu, The role of driving factors in historical and projected carbon dynamics of upland ecosystems in Alaska. *Ecol. Appl.* **28**, 5–27 (2018).

59. B. D. Amiro, A. Cantin, M. D. Flannigan, W. J. de Groot, Future emissions from Canadian boreal forest fires. *Can. J. For. Res.* **39**, 383–395 (2009).

60. X. Yue, L. J. Mickley, J. A. Logan, R. C. Hudman, M. V. Martin, R. M. Yantosca, Impact of 2050 climate change on North American wildfire: Consequences for ozone air quality. *Atmos. Chem. Phys.* **15**, 10033–10055 (2015).

61. Y. Boulanger, S. Gauthier, P. J. Burton, A refinement of models projecting future Canadian fire regimes using homogeneous fire regime zones. *Can. J. For. Res.* **44**, 365–376 (2014).

62. E. S. Hope, D. W. McKenney, J. H. Pedlar, B. J. Stocks, S. Gauthier, Wildfire suppression costs for Canada under a changing climate. *PLOS ONE* **11**, e0157425 (2016).

63. E. S. Euskirchen, A. D. McGuire, T. S. Rupp, F. S. Chapin, J. E. Walsh, Projected changes in atmospheric heating due to changes in fire disturbance and the snow season in the western Arctic, 2003–2100. *J. Geophys. Res. Biogeo.* **114**, G04022 (2009).

64. A. M. Young, P. E. Higuera, P. A. Duffy, F. S. Hu, Climatic thresholds shape northern high-latitude fire regimes and imply vulnerability to future climate change. *Ecography* **40**, 606–617 (2017).

65. X. J. Walker, J. L. Baltzer, L. L. Bourgeau-Chavez, N. J. Day, W. J. De groot, C. Dieleman, E. E. Hoy, J. F. Johnstone, E. S. Kane, M. A. Parisien, S. Potter, B. M. Rogers, M. R. Turetsky, S. Veraverbeke, E. Whitman, M. C. Mack, “ABoVE: Synthesis of Burned and Unburned Forest Site Data, AK and Canada, 1983-2016” (ORNL DAAC, 2019); https://doi.org/10.3334/ORNLDAAC/1744.

66. M. L. Goulden, A. M. S. Mcmillan, G. C. Winston, A. V. Rocha, K. L. Manies, J. W. Harden, B. P. Bond-Lamberty, Patterns of NPP, GPP, respiration, and NEP during boreal forest succession. *Glob. Chang. Biol.* **17**, 855–871 (2011).

67. J. M. Omernik, Perspectives on the nature and definition of ecological regions. *Environ. Manag.* **34**, S27–S38 (2004).

68. E. S. Kasischke, D. Williams, D. Barry, Analysis of the patterns of large fires in the boreal forest region of Alaska. *Int. J. Wildland Fire.* **11**, 131 (2002).

69. L. DeWilde, F. S. Chapin, Human impacts on the fire regime of interior Alaska: Interactions among fuels, ignition sources, and fire suppression. *Ecosystems* **9**, 1342–1353 (2006).

70. R. D. Ottmar, D. V. Sandberg, C. L. Riccardi, S. J. Prichard, An overview of the Fuel Characteristic Classification System — Quantifying, classifying, and creating fuelbeds for resource planningThis article is one of a selection of papers published in the Special Forum on the Fuel Characteristic Classification System. *Can. J. For. Res.* **37**, 2383–2393 (2007).

71. R. Gelaro, W. McCarty, M. J. Suárez, R. Todling, A. Molod, L. Takacs, C. A. Randles, A. Darmenov, M. G. Bosilovich, R. Reichle, K. Wargan, L. Coy, R. Cullather, C. Draper, S. Akella, V. Buchard, A. Conaty, A. M. da Silva, W. Gu, G.-K. Kim, R. Koster, R. Lucchesi, D. Merkova, J. E. Nielsen, G. Partyka, S. Pawson, W. Putman, M. Rienecker, S. D. Schubert, M. Sienkiewicz, B. Zhao, The Modern-Era Retrospective Analysis for Research and Applications, Version 2 (MERRA-2). *J. Climate* **30**, 5419–5454 (2017).

72. R. D. Field, A. C. Spessa, N. A. Aziz, A. Camia, A. Cantin, R. Carr, W. J. de Groot, A. J. Dowdy, M. D. Flannigan, K. Manomaiphiboon, F. Pappenberger, V. Tanpipat, X. Wang, Development of a Global Fire Weather Database. *Nat. Hazards Earth Syst. Sci.* **15**, 1407–1423 (2015).

73. N. Gorelick, M. Hancher, M. Dixon, S. Ilyushchenko, D. Thau, R. Moore, Google Earth Engine: Planetary-scale geospatial analysis for everyone. *Remote Sens. Environ.* **202**, 18–27 (2017).

74. M. Flannigan, C. Vanwagner, Climate change and wildfire in Canada. *Can. J. For. Res.* **21**, 66–72 (1991).

75. D. Bachelet, J. Lenihan, R. Neilson, R. Drapek, T. Kittel, Simulating the response of natural ecosystems and their fire regimes to climatic variability in Alaska. *Can. J. For. Res.* **35**, 2244–2257 (2005).

76. S. Gauthier, P. Bernier, T. Kuuluvainen, A. Z. Shvidenko, D. G. Schepaschenko, Boreal forest health and global change. *Science* **349**, 819–822 (2015).

77. Y. Boulanger, M.-A. Parisien, X. Wang, Model-specification uncertainty in future area burned by wildfires in Canada. *Int. J. Wildland Fire* **27**, 164–175 (2018).

78. M. Flannigan, K. Logan, B. Amiro, W. Skinner, B. Stocks, Future area burned in Canada. *Clim. Change* **72**, 1–16 (2005).

79. IPCC, 2021: Summary for policymakers, in *Climate Change 2021: The Physical Science Basis. Contribution of Working Group I to the Sixth Assessment Report of the Intergovernmental Panel on Climate Change*, V. MassonDelmotte, P. Zhai, A. Pirani, S. L. Connors, C. Péan, S. Berger, N. Caud, Y. Chen, L. Goldfarb, M. I. Gomis, M. Huang, K. Leitzell, E. Lonnoy, J. B. R. Matthews, T. K. Maycock, T. Waterfield, O. Yelekçi, R. Yu, B. Zhou, Eds. (Cambridge Univ. Press, 2021).

80. K. Gillingham, J. H. Stock, The cost of reducing greenhouse gas emissions. *J. Econ. Perspect.* **32**, 53–72 (2018)

81. D. Ray, "Lazard's levelized cost of energy analysis—Version 13.0, 20" (Lazard, 2019).

82. National Academies of Sciences, "Negative emissions technologies and reliable sequestration: A research agenda" (National Academies of Sciences, 2018).
